# NFIB Mediates BRN2 Driven Melanoma Cell Migration and Invasion Through Regulation of EZH2 and MITF

**DOI:** 10.1016/j.ebiom.2017.01.013

**Published:** 2017-01-16

**Authors:** Mitchell E. Fane, Yash Chhabra, David E.J. Hollingsworth, Jacinta L. Simmons, Loredana Spoerri, Tae Gyu Oh, Jagat Chauhan, Toby Chin, Lachlan Harris, Tracey J. Harvey, George E.O. Muscat, Colin R. Goding, Richard A Sturm, Nikolas K. Haass, Glen M. Boyle, Michael Piper, Aaron G. Smith

**Affiliations:** a The School of Biomedical Sciences, The University of Queensland, Brisbane, QLD 4072, Australia; b School of Biomedical Sciences, Institute of Health and Biomedical Innovation at the Translational Research Institute, Queensland University of Technology, Woolloongabba, QLD 4102, Australia; c Cancer Drug Mechanisms Group, Department of Cell and Molecular Biology, QIMR Berghofer Medical Research Institute, Herston, Queensland 4006, Australia; d The University of Queensland Diamantina Institute, The University of Queensland, Brisbane, QLD, Australia; e Institute for Molecular Bioscience, The University of Queensland, St. Lucia, QLD 4072, Australia; f Ludwig Institute for Cancer Research, Nuffield Department of Medicine, University of Oxford, Headington, Oxford OX3 7DQ, UK; g Dermatology Research Centre, The University of Queensland, School of Medicine, Translational Research Institute, Brisbane, QLD 4102, Australia; h Queensland Brain Institute, The University of Queensland, Brisbane, QLD 4072, Australia

**Keywords:** Melanoma, Metastasis, Invasion, BRN2, NFIB, Epigenetic

## Abstract

While invasion and metastasis of tumour cells are the principle factor responsible for cancer related deaths, the mechanisms governing the process remain poorly defined. Moreover, phenotypic divergence of sub-populations of tumour cells is known to underpin alternative behaviors linked to tumour progression such as proliferation, survival and invasion. In the context of melanoma, heterogeneity between two transcription factors, BRN2 and MITF, has been associated with phenotypic switching between predominantly invasive and proliferative behaviors respectively. Epigenetic changes, in response to external cues, have been proposed to underpin this process, however the mechanism by which the phenotypic switch occurs is unclear. Here we report the identification of the NFIB transcription factor as a novel downstream effector of BRN2 function in melanoma cells linked to the migratory and invasive characteristics of these cells. Furthermore, the function of NFIB appears to drive an invasive phenotype through an epigenetic mechanism achieved via the upregulation of the polycomb group protein EZH2. A notable target of NFIB mediated up-regulation of EZH2 is decreased MITF expression, which further promotes a less proliferative, more invasive phenotype. Together our data reveal that NFIB has the ability to promote dynamic changes in the chromatin state of melanoma cells to facilitate migration, invasion and metastasis.

## Highlights

• NFIB mediates a slow cycling, highly invasive/migratory melanoma cell phenotype downstream of BRN2.
• NFIB increases EZH2 expression downstream of BRN2, which further decreases MITF levels.
• NFIB expression is defined by an invasive gene signature and colocalises with BRN2 in primary and metastatic human melanoma tumours.

Melanoma is a heterogeneous cancer, made up of many cellular populations that differ in their ability to induce tumour growth or invasion throughout the body (metastasis). These populations have been found to switch back and forth to drive invasion and progression. This process appears to be controlled by an inverse axis between two genes, MITF and BRN2. BRN2 drives metastatic spread, but the process by which it acts is not well characterized and cannot be targeted clinically. This study has uncovered a role for the gene NFIB in driving invasion downstream of BRN2. Importantly, it appears to drive this process through EZH2, which can be targeted therapeutically to reduce metastasis.

## 1 Introduction

The underlying genetic and phenotypic heterogeneity associated with melanoma tumours has resulted in a limited range of therapeutic options as the disease progresses towards metastasis (Goodall et al., 2008, Quintana et al., 2010, Thurber et al., 2011). Such heterogeneity is not only defined in terms of mutations in tumour suppressor and proto-oncogenes between patients, but is further seen in individual tumour subpopulations that differ in terms of phenotypic characteristics such as proliferation and invasion. This heterogeneity has previously been explained by irreversible genetic models such as clonal evolution (Nowell, 1976) and the cancer stem cell model (Reya et al., 2001), but much evidence now suggests that the epigenetic and molecular changes associated with these phenotypes are highly reversible and switch in response to changes in the tumour microenvironment (Hoek et al., 2008, Thurber et al., 2011, Wellbrock and Arozarena, 2015).

MITF and BRN2 have been found to mark two distinct subpopulations of cells within melanoma tumours that drive opposing phenotypes (Goodall et al., 2008, Wellbrock and Arozarena, 2015). MITF expressing cell types are characterized as highly proliferative and more differentiated, while BRN2 populations adopt a highly invasive, stem-cell like appearance (Goodall et al., 2008). While the idea of phenotype switching and mutual exclusivity between these two cell types is debatable (Chapman et al., 2014, Haass et al., 2014), it is clear that BRN2 drives an invasive melanoma cell phenotype (Arozarena et al., 2011, Boyle et al., 2011, Kobi et al., 2010). Despite this, very little is known about downstream effector BRN2 targets that may drive this undifferentiated migratory cell type.

The nuclear factor one (NF1) family consists of four site-specific transcription factors (*NfiA*, *NfiB*, *NfiC*, and *NfiX*) that play a large role in the development of a number of organs systems such as the lung and brain (Grunder et al., 2002, Harris et al., 2015, Hsu et al., 2011, Steele-Perkins et al., 2005), and is upregulated in many epithelial type cancers (Dooley et al., 2011, Moon et al., 2011). While the expression of NFIB has not been reported in melanocytic cells, recent findings have demonstrated that NFIB governs epithelial-melanocyte stem cell behavior in a shared niche (Chang et al., 2013). Interestingly BRN2 has been proposed to maintain a less differentiated, stem cell-like phenotype in melanoblasts and a subset of melanoma cells (Cook et al., 2003, Cook and Sturm, 2008, Goodall et al., 2008). Accordingly, we chose to examine the expression and function of NFIB in the context of BRN2 function in primary melanocytic cells and melanoma cells to determine if this transcription factor plays a role in BRN2 mediated phenotype switching.

## 2 Materials and Methods

### 2.1 Ethics Statement

Animal studies were performed in strict accordance with the recommendations in the Australian Code for the Care and Use of Animals for Scientific Purposes 8th Edition (2013), of the National Health and Medical Research Council of Australia. All protocols were reviewed and approved by the QIMR Berghofer Medical Research Institute Animal Ethics Committee (QIMR-AEC). All mice were housed in a specific pathogen free (SPF) facility, with 12 h light/dark cycle and continual access to food and water. Melanoma biopsies were collected with informed patient consent, under a protocol approved by the Queensland Institute of Medical Research and Princess Alexandra Hospital Human Ethics Committees. The study was conducted according to the Declaration of Helsinki Principles.

### 2.2 Cell Culture and Transfection Analysis

A2058, MM96L, HT144, A11, MM418.5c, and MM537 human melanoma cell lines were cultured in RPMI media supplemented with 5% foetal bovine serum, 2 mM L-glutamine, and 5 mg/ml penicillin/streptomycin. QF human melanoblast cell strains (MB) were cultured as previously described (Cook et al., 2003). BRN2, MITF, and NFIB siRNA transfection in human melanoma cell lines were performed in 6 well dishes as previously described (Thurber et al., 2011). The melanoblast to melanocyte (MC) morphology differentiation (MB:MC) was performed as previously described (Cook et al., 2003). The EZH2 inhibitor GSK343 (Sigma Aldrich) was dissolved in DMSO and was used at a concentration of 0.1 μM, 1 μM and 10 μM. TetOff stable cells were cultured in 100 ng/ml of doxycycline, which was removed at the start of experiments to induce expression of the transgene. All three parental lines tested were treated with doxycycline at 100 ng/ml to test for any off target effects and did not show any difference in the basal levels of BRN2, NFIB, EZH2, GAPDH or any other genes of interest (data not shown).

### 2.3 Melanoma Sphere/Spheroid Formation and Invasion Assays

A2058 parental, BRN2, MITF and a 50:50 co-culture of A2058-GFP and A2058-BRN2 cell lines were cultured as melanoma spheres and sectioned as previously described (Thurber et al., 2011). Melanoma spheroids were prepared as described (Beaumont et al., 2015, Smalley et al., 2008, Spoerri et al., 2017). Both sphere and spheroid models mimic in vivo tumour architecture and microenvironment and are used for investigating growth, invasion and viability of melanoma cells (Beaumont et al., 2014, Santiago-Walker et al., 2009). Melanoma spheroids were formed using A2058 empty, BRN2, MITF and NFIB cells and invasion assays were performed as previously described (Haass et al., 2014). Images were quantified using Image J software (http://rsb.info.nih.gov/ij) to calculate the area covered by the invading cells relative to the spheroid and overall spheroid growth.

### 2.4 Western Blot

Western Blot analysis was performed using techniques described previously (Cook et al., 2003). Immunoblots were probed using rabbit monoclonal BRN2 antibody D2C1L (Cell Signaling #12137), rabbit monoclonal NFIB antibody (Sigma Aldrich; HPA003956), rabbit monoclonal MITF D567V (Cell Signaling #12590), mouse monoclonal EZH2 (Active Motif #39639), and Rabbit monoclonal H3K27 tri-methyl C36B11 (Cell Signaling #9733), with anti-GAPDH 6C5 (Santa Cruz #sc-32233) used as a loading control.

### 2.5 Xenograft Studies

For tumourigenicity studies, 2.0 × 10^6^ cells in 50 μl of RPMI-1640/10% FCS were injected intra-dermally into each of two sites of five five-week old male immunocompromised BALB/c *Foxn1*^*nu*^ mice. All mice were monitored daily and tumour volume measured at least twice weekly, recorded using digital calipers and expressed as mm^3^ according to the formula A × b × b × 0.5 where A the length and b the measured breadth of the tumour. Mice were also assessed for clinical signs according to a QIMR-AEC approved clinical score sheet for distress during the period of the experiment to determine whether tumour burden was causing distress to the mice to a degree and to where they should be euthanized.

### 2.6 Statistical Analysis

Densitometry analysis was performed on Western Blots to quantify the size and intensity of the band relative to a control sample to give overall fold change and were normalized using GAPDH as a loading control. Wound healing assays, luciferase activity, and invasion/spheroid growth assays were analyzed using a two-way ANOVA with a Tukey's post-hoc test, with data presented as the mean  ± SEM. A one-way ANOVA with Dunnett's multiple comparisons test was performed on quantitative real time samples.

## 3 Results

### 3.1 BRN2 and NFIB Expression in Melanocytic Cells

**Fig. 1:**
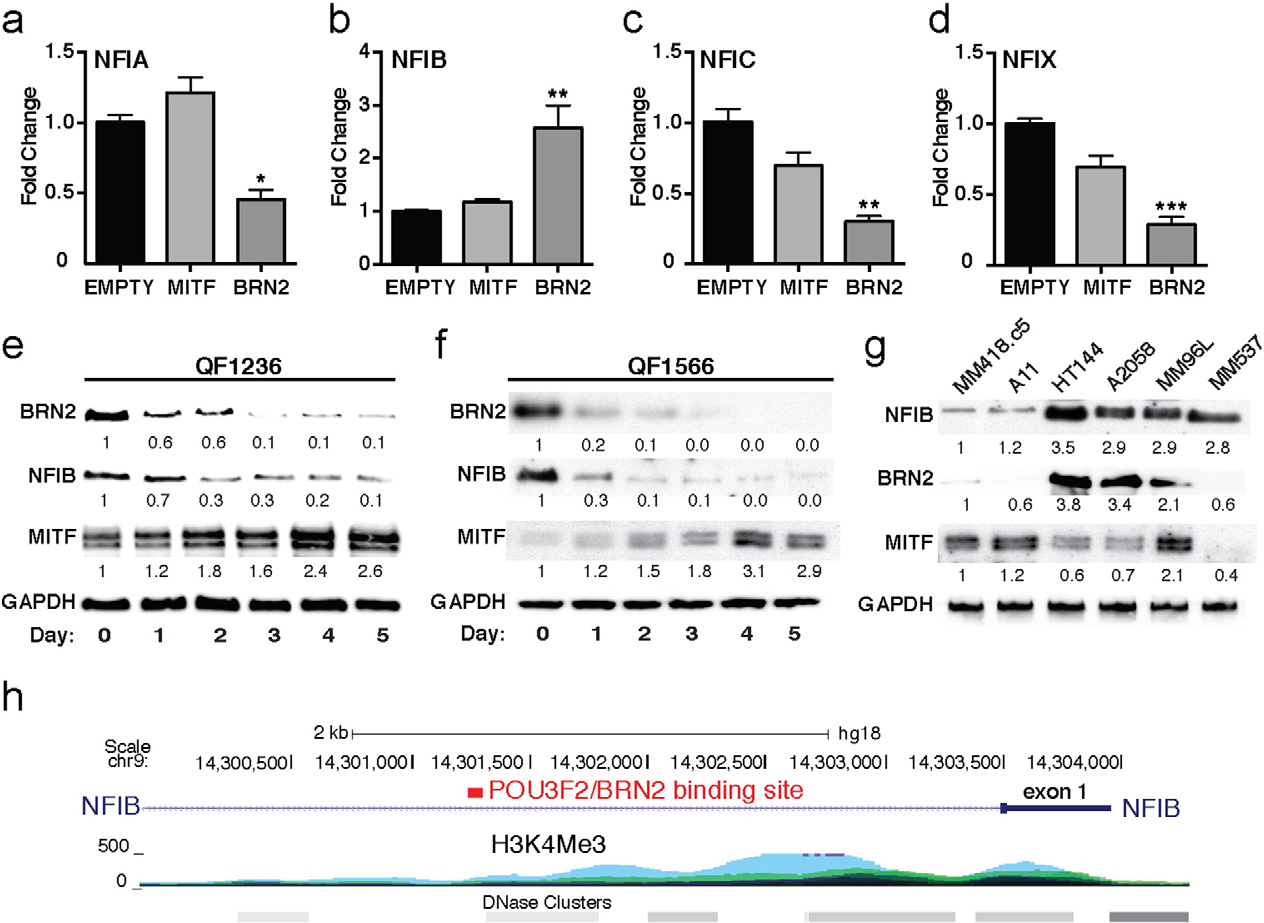
NFIB expression correlates with BRN2 in melanocytic and melanoma cells. (A–D) QPCR analysis on A2058 melanoma cells following lentiviral transduction to produce stable over-expression of MITF or BRN2, investigating *NFIA*, *NFIB*, *NFIC*, and *NFIX* expression. Data represented as fold change relative to the empty vector control and normalized to B2M gene. (E–F) Western Blot analysis on neonatal foreskin-derived QF1236 and QF1566 primary human melanoblast cells induced to differentiate into pigmented melanocytes over a 5-day period. Antibodies were used against BRN2, NFIB, MITF, and GAPDH. (G) Whole cell lysates from six human melanoma cell lines immunoblotted for NFIB, BRN2, and MITF. (H) ChIP-ChIP analysis data (Kobi et al., 2010) in 501 Mel human melanoma cells investigating BRN2 binding to chromatin regions, reveals BRN2 binds to a 2Kb intronic region located upstream of the NFIB promoter. *: *P* < 0.05, **: *P* < 0.01, ***: *P* < 0.001. Data representative of three independent experiments. Band expression intensity of Western Blots was normalized to the first lane (GAPDH used as a loading control) using ImageJ software and indicated below each blot. Data from (A–D) is represented as the mean ± SEM and analysed using a one-way ANOVA with Dunnett's multiple comparisons test. See also Fig. S3.

The expression and function of the nuclear factor one (NFI) family of transcription factors have not been reported previously in melanocytic cells. Initially, we were interested in the potential role these genes may play in the context of the BRN2-MITF expression axis that has been proposed to drive melanoma progression. A2058 human melanoma cells engineered to over-express either BRN2 or MITF were analyzed by qRT-PCR for expression of all four members of the NFI gene family, *NFIA*, *NFIB*, *NFIC* and *NFIX* (Fig. 1A–D). MITF overexpression was found to have only a modest, non-significant effect on the expression of these genes at the transcript level when compared with empty vector control cells. BRN2 overexpression induced a significant decrease in *NFIA*, *NFIC*, and *NFIX* (Fig. 1A, C, and D), but interestingly resulted in a significant increase in *NFIB* expression (Fig. 1B). Subsequently, we chose to examine the expression of NFIB in primary human melanoblast (MB) cells induced to differentiate into fully pigmented mature melanocytes (MC) (Cook et al., 2003). Three independent MB cell lines derived from neonatal human foreskin were cultured until confluent and induced to differentiate via treatment with conditioned media over a 5 day period, with protein lysates taken initially (day 0) and at 24 h intervals following treatment (Cook et al., 2003). BRN2 and MITF protein levels were found to be inversely correlated over the course of differentiation, with BRN2 levels decreasing as differentiation progressed (Figs. 1E, F, and S3A). NFIB levels followed a similar trend, gradually decreasing ten-fold as differentiation progressed (Figs. 1E, F, and S3A).

As BRN2 and MITF have been found to exhibit a predominantly inverse expression pattern in melanoma cells which promotes switching between invasive/migratory and proliferative phenotypes (Goodall et al., 2008, Hoek and Goding, 2010), we chose to examine the expression of BRN2, MITF and NFIB proteins in six melanoma cell lines. Expression levels were normalized to the MM418c5 line. NFIB expression was found to be significantly higher in four of the six lines (2.8–3.5 fold) with considerably lower expression seen in the MM418c5 and A11 melanoma lines (Fig. 1G). Interestingly, low NFIB lines also expressed negligible BRN2 protein but high MITF levels (Fig. 1G, lanes 1–2). Moreover, of the four lines expressing high NFIB levels, three lines (HT144, A2058 and MM96L) expressed high levels of BRN2 (Fig. 1G). MITF expression in the NFIB high lines was generally lower than the MM418.c5 or A11 lines which have low NFIB expression. One line, MM537 expressed high levels of NFIB despite negligible expression of either BRN2 or MITF transcription factors (0.6 and 0.4 fold relative to MM418.c5 respectively).

Together this data suggests a relationship between NFIB and BRN2 expression in melanoma cells at both the protein and transcript levels. To further explore this possibility we examined a published ChIP-chip data set that examined BRN2 promoter/enhancer occupancy in 501Mel cells (Kobi et al., 2010). This analysis revealed enrichment of BRN2 binding within the first intron of the NFIB gene locus (Fig. 1H), further suggesting that BRN2 may be able to directly bind NFIB and regulate its expression in melanoma cells.

### 3.2 BRN2 Regulates NFIB and EZH2 Expression In-vitro

**Fig. 2:**
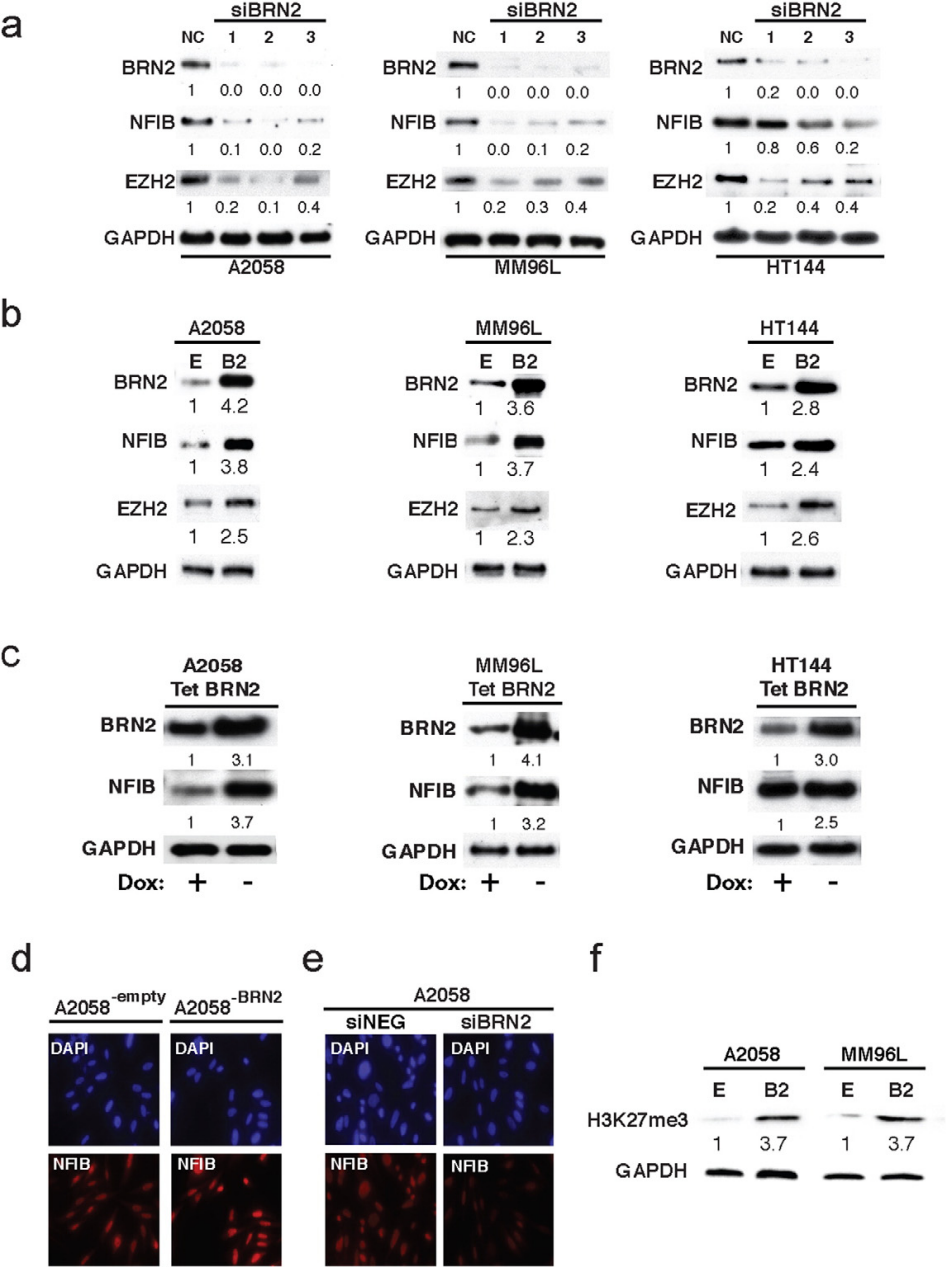
BRN2 positively regulates NFIB and EZH2 expression. (A) Cell lysates from A2058, MM96L, and HT144 human melanoma cells treated with three different siRNA directed against BRN2 were analyzed by Western Blot with antibodies against BRN2, NFIB, EZH2, and GAPDH (B) Cell lysates from A2058, MM96L, and HT144 human melanoma cells treated with lentivirus to create stable overexpression of BRN2 were analyzed by Western Blot with antibodies against BRN2, NFIB, EZH2, and GAPDH (C) Cell lysates from doxocycline-off inducible BRN2 expressing A2058, MM96L, and HT144 cells treated with and without dox for 48 h were analyzed by Western Blot with antibodies against BRN2, NFIB, EZH2, and GAPDH. (D) NFIB immunofluorescence (red) on A2058 parental cells grown on coverslips and treated with siRNA directed against BRN2. DAPI used to stain cell nuclei. (E) NFIB immunofluorescence (red) on A2058 BRN2 over-expressing cells. DAPI used to stain cell nuclei. (F) Cell lysates from A2058 and MM96L melanoma cells stably over-expressing BRN2 were analyzed by Western Blot for H3K27 tri methylation status (EZH2 global methylation marker). Band expression intensity of Western Blots was normalized to the first lane (GAPDH used as a loading control) using ImageJ software and indicated below each blot. All data representative of three independent experiments.

To further investigate the potential relationship between NFIB and BRN2, the effect of gain or loss of BRN2 expression in a number of different melanoma cell lines was tested. Firstly, siRNA knockdown of BRN2 in high NFIB expressing lines (A2058, MM96L and HT144) produced a considerable reduction in NFIB expression to levels of approximately 80% fold or less in A2058 and MM96L melanoma cells and a modest but consistent reduction in the HT144 cells (20%, 40% and 80%) across the three independent siRNA treatment groups (Fig. 2A). The epigenetic modulator EZH2 was also investigated as it has recently been linked to melanoma invasion and metastasis (Zingg et al., 2015), and has been previously identified as a target of NFIB in neuronal cell lineages (Piper et al., 2014). EZH2 expression was found to decrease in response to BRN2 siRNA knockdown in all three melanoma cell lines, reduced to levels of 0.4 fold or lower across all three lines tested compared to the negative siRNA control cells (Fig. 2A).

Conversely, constitutive BRN2 overexpression was also performed in these same cell lines and revealed a commensurate increase in both NFIB (3.8, 3.7, 2.4 fold respectively) and EZH2 (2.5, 2.3, 2.6 fold respectively) expression across all three melanoma cell lines (Fig. 2B). Similarly, doxycycline controlled up-regulation of BRN2 in these cells (TET-OFF) promoted a reciprocal increase in NFIB expression to levels of 3.7, 3.2, and 2.5 fold across all three lines (Fig. 2C). Increased or decreased NFIB expression in response to BRN2 gain or loss of function was also confirmed using immunofluorescence (Fig. 2D and E), further supporting a potential role for BRN2 in regulating the expression of both genes.

As EZH2 induces Histone-H3 tri-methylation at Lysine 27 (H3-K27me3) we analyzed our protein lysates for global changes in H3-K27me3 levels to determine if BRN2 increased EZH2 levels correlate with increased activity of the protein. It was found that H3-K27me3 levels increased to 3.7 fold in both A2058^− BRN2^ and MM96L^− BRN2^ cells compared to the respective empty vector control lines (Fig. 2F).

### 3.3 Effect of Modulating MITF Expression on NFIB Expression in Melanoma Cells

Similar knockdown and overexpression experiments targeting MITF in these cell lines revealed an inverse effect on NFIB and EZH2 expression levels to that mediated by BRN2. Specifically, over-expression of MITF was found to decrease both NFIB and EZH2 levels, while MITF knock-down increased NFIB and EZH2, although its effect on NFIB levels in MM96L and HT144 cell lines were not as pronounced as those seen in A2058’s (Fig. S4A and S4B). Furthermore, MITF over-expression in both A2058 and MM96L cells resulted in a global reduction on H3-K27me3 levels (Fig. S4C).

### 3.4 BRN2 Regulates NFIB Expression Within 3D Melanoma Spheres

Melanoma spheres provide a useful *in vitro* 3D model of melanoma to better recapitulate an *in vivo* tumour microenvironment and architecture (Fang et al., 2005). Moreover, spheres have been found to recapitulate the BRN2-MITF heterogeneity observed *in vivo* in contrast to that seen using 2D culture and some spheroid models (Thurber et al., 2011). Melanoma spheres were generated using A2058^− empty^, A2058^− BRN2^, or A2058^− MITF^ stable cell lines. Analysis of empty spheres reveals that BRN2 predominantly localizes in cell populations in the periphery, while MITF expression appears heterogeneous throughout the sphere in A2058^− empty^ cells (Fig. S7A). Similar patterns were detected using the parental A2058 cells (data not shown). Co-staining of BRN2 and MITF reveals a large number of cells exclusively expressing either BRN2 or MITF (Fig. S7A), whereas staining of A2058 cells grown as a 2D mono-layer predominately shows co-localization between the two transcription factors in the majority of cells (Thurber et al., 2011). Western Blot analysis of A2058^− BRN2^ and A2058^− MITF^ spheres confirms that they are inversely regulated upon overexpression (Fig. S7B).

NFIB positive cells were found to exhibit a similar distribution to BRN2 positive populations within the spheres (Fig. S7C) and were predominately localized in the periphery of parental spheres generated using A2058^− empty^ (Fig. S7C upper panel). Furthermore, A2058^− BRN2^ spheres resulted in an increase in NFIB positive cells, which were distributed evenly throughout the sphere (Fig. S7C, middle panel). The increase in NFIB levels in the A2058^− BRN2^ spheres was also validated by Western Blot analysis (Fig. S7C). Additionally, A2058^− MITF^ overexpressing spheres showed a slight decrease in NFIB protein expression (30% reduction), with localization in the spheres now randomized throughout much lower and more randomized throughout (Fig. S7C, lower panel).

### 3.5 Both BRN2 and NFIB Drive a Highly Migratory Melanoma Cell Phenotype

**Fig. 3:**
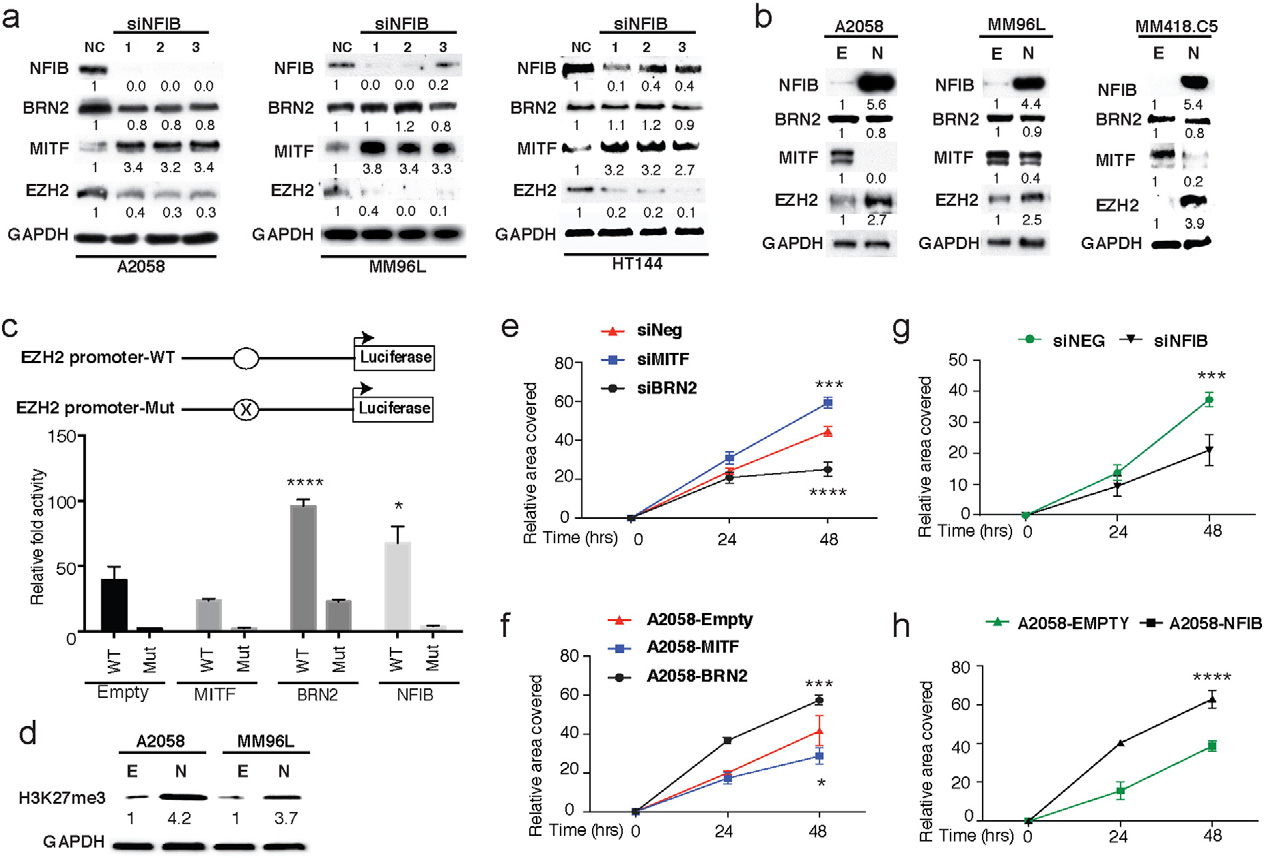
NFIB manipulation increases migration and EZH2 expression and decreases MITF expression. (A) Cell lysates from A2058, MM96L, and HT144 human melanoma cells treated with three different siRNA directed against NFIB were analyzed by Western Blot with antibodies against BRN2, NFIB, EZH2, MITF, and GAPDH. (B) Cell lysates from A2058, MM96L, and HT144 human melanoma cells treated with lentivirus to create stable overexpression of NFIB were analyzed by Western Blot with antibodies against BRN2, NFIB, EZH2, MITF and GAPDH. (C) A2058 stables for MITF, BRN2, NFIB, and empty control cells transfected with two luciferase reporter constructs; A wild type construct containing a region of the EZH2 promoter containing the NFIB putative binding site (WT) and a mutant construct with the NFI binding site mutated out (Mut). Data represented as relative luciferase fold activity following normalisation against the empty-Mut. (D) Cell lysates from stable NFIB overexpressing A2058 and MM96L melanoma cells were analyzed by Western Blot for H3K27 tri methylation status (EZH2 global methylation marker). (E) Quantification of wound healing assay performed on A2058 human melanoma cells treated with siRNA against BRN2, MITF or a scrambled control 24 h prior to wound initiation. (F) Quantification of wound healing assay performed in A2058 stable BRN2, MITF and empty control cells. (G) Quantification of wound healing assay performed in A2058 human melanoma cells treated with siRNA against BRN2, MITF or a scrambled control 24 h prior to commencement of the experiment. (H) Quantification of wound healing assay performed in A2058 stable NFIB and empty control cells. Data represented as the mean ± SEM. *: *P* < 0.05, ***: *P* < 0.001, ****: *P* < 0.0001. A two-way ANOVA with a Tukey's post-hoc test was performed in (C) and (E–H). All data representative of three independent experiments. See also Figs. S1, S2, S3 and S5.

**Fig. 4:**
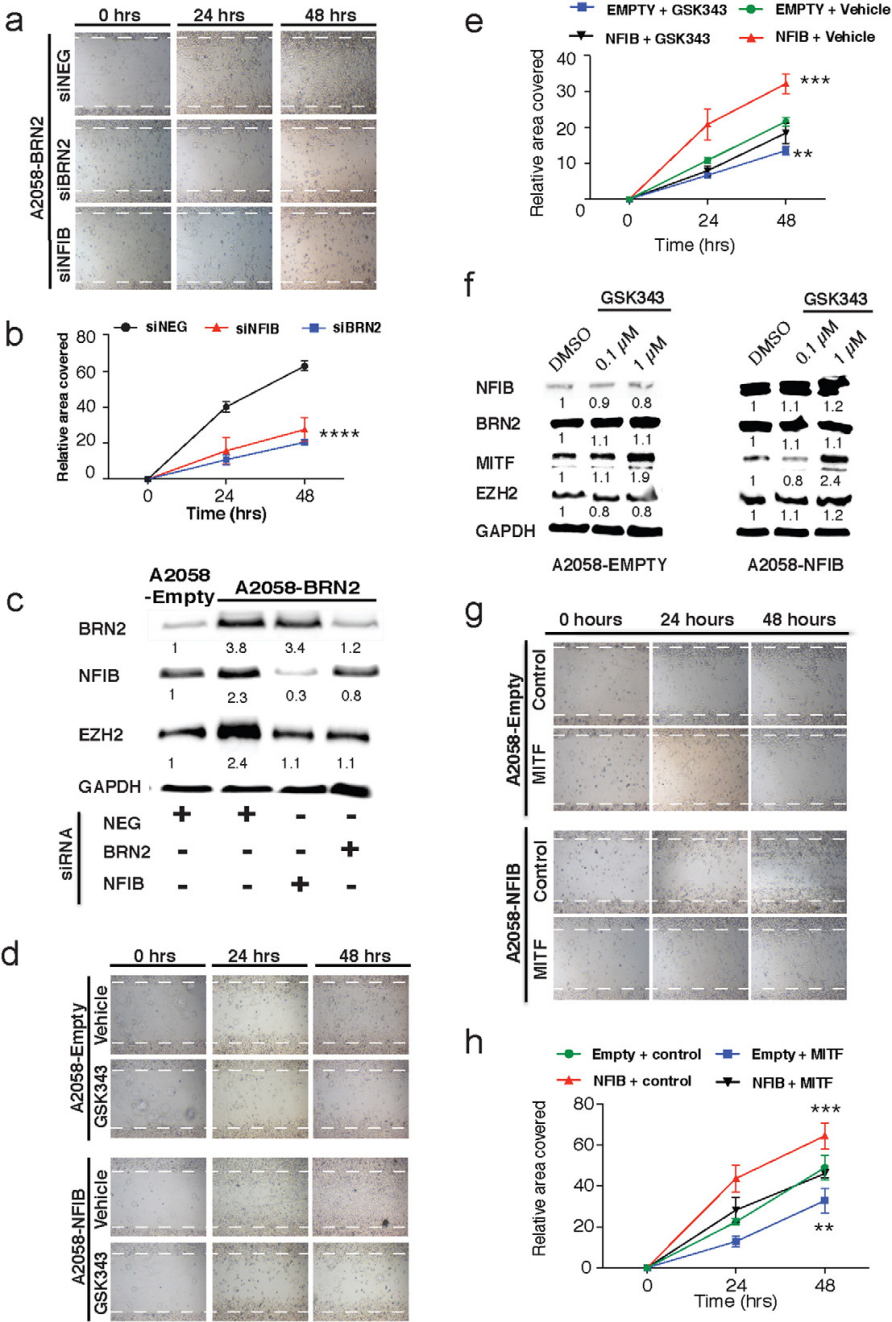
NFIB drives migration downstream of BRN2 through interactions with EZH2. (A–B) Lightphase images and quantification of wound healing assays performed in A2058 stable BRN2 cells treated with siRNA against BRN2, NFIB or a scrambled control 24 h prior to wound initiation (0 h), with images taken at 0, 24, and 48 h. (C) Cell lysates from A2058 BRN2 stable melanoma cells treated with siRNA against BRN2, NFIB or a scrambled control were analyzed by Western Blot with antibodies against BRN2, NFIB, EZH2, and GAPDH. (D-E) Lightphase images and quantification of wound healing assays performed in A2058 stable NFIB cells treated with EZH2 inhibitor GSK343 at 1 μM or a vehicle control (DMSO) 24 h prior to wound inititation (0 h), with images taken at 0, 24, and 48 h. (F) Cell lysates from A2058 NFIB stable melanoma cells treated with GSK343 at 0.1 μM, 1 μM or a vehicle control (DMSO) were analyzed by Western Blot with antibodies against BRN2, NFIB, EZH2, MITF and GAPDH. (G-H) Lightphase images and quantification of wound healing assays performed in A2058 stable NFIB melanoma cells treated with MITF or empty control lentivirus 24 h prior to commencement of the experiment, with images taken at 0, 24, and 48 h. Data represented as the mean ± SEM and analysed with two-way ANOVA with a Tukey's post hoc test. **: *P* < 0.01, ***: *P* < 0.001, ****: *P* < 0.0001. All data representative of three independent experiments. Band expression intensity of Western Blots was normalized to the first lane (GAPDH used as a loading control) using ImageJ software and indicated below each blot. See also Figs. S2 and S6.

Wound-healing assays were first performed in BRN2 and MITF overexpressing cells (Figs. 3F and S5A–C) and in cells treated with pooled siRNA against BRN2 and MITF (Figs. 3E and S5D–F) to assess migration. Quantification of wound repopulation, shown below the respective images, reveals that BRN2 overexpression significantly increased the cells ability to migrate and fill the scratch, while knockdown of BRN2 significantly impaired migration (Figs. 3E, 4F and S5). Conversely, the opposite trend was observed for MITF where migration was significantly impaired by over-expression and enhanced by siRNA knock-down (Figs. 3E, 4F, and S5). Modulation of NFIB expression induced a very similar migration phenotype to BRN2 whereby a significantly increased migratory capacity was seen in response to over-expression of NFIB within wound healing assays (Figs. 3H, S1B, S1D, S1F and S1H), while siRNA knock-down significantly decreased melanoma cell migration rates (Figs. 3G, S1A, S1C, S1E and S1G).

### 3.6 NFIB Directly Increases EZH2 Expression

To extend upon the finding that BRN2 drives NFIB expression, we wanted to further determine if the up-regulation of EZH2 in response to BRN2 over-expression was mediated by NFIB. Firstly, we confirmed that while siRNA knock-down of NFIB had a negligible effect on BRN2 expression, EZH2 levels were significantly reduced in all lines tested using three independent siRNAs targeting NFIB (Fig. 3A). Conversely, over-expression of NFIB in A2058, MM96L (relatively high NFIB lines), MM418.5c, and A11 cell lines (both found to express minimal endogenous NFIB protein) induced a commensurate up-regulation in EZH2 (Figs. 3B and S3G). Notably, modulation of NFIB had a substantial inverse effect on MITF expression in these studies, with MITF levels increasing or decreasing following NFIB knockdown or overexpression respectively (Figs. 3A and 4B).

Next, the activity of an EZH2 promoter-luciferase reporter (WT), or a mutant version in which the NFIB consensus sites have been mutated (Mut), was determined in the A2058 over-expression lines (empty, MITF, BRN2 and NFIB). The immediate EZH2 promoter (WT) was significantly more active in cells over-expressing BRN2 (*P* < 0.0001) and NFIB (*P* < 0.05) compared to the empty control cells (Fig. 3C). Interestingly, mutation of a previously identified NFIB binding site in this region (Piper et al., 2014) dramatically reduced cis-activity in all lines tested including BRN2, suggesting that NFIB binding is important in driving EZH2 promoter driven expression within this region downstream of BRN2 (Fig. 3C). While activity of the mutant reporter was lower than the WT in the BRN2 over-expressing cells, this reporter was considerably more active in these cells when compared with the other stable cell lines, suggesting BRN2 might also be able to regulate the activity of this region independently of NFIB, either directly or indirectly.

To further assess whether NFIB mediated increases in EZH2 protein levels also correlated with an increase in EZH2 activity, global levels of the methylation marker H3-K27me3 were investigated via Western Blot analysis and were found to increase in both A2058^− NFIB^ and MM96L^− NFIB^ cells compared to the respective empty vector control cells (Fig. 3D).

### 3.7 NFIB Negatively Regulates MITF Expression in Melanoma Cells

In addition to upregulating EZH2 expression when overexpressed, modulation of NFIB levels had a striking effect on MITF expression with a clear inverse relationship predominating (Figs. 3A, 4B and S3G). To examine this effect further, a luciferase reporter driven by the immediate 1.8 kb region extending immediately upstream of the MITF-M transcriptional start site was transfected into A2058^− empty^, A2058^− BRN2^ and A2058^− NFIB^ melanoma cell lines (Fig. S2E). The activity of this region was found to be significantly lower in both BRN2 (*P* < 0.001) and NFIB (*P* < 0.0001) over-expressing cells compared to the empty control. Furthermore, the cis-activity of the MITF promoter region was significantly elevated to similar levels in all three lines when cells were treated with the EZH2 inhibitor GSK343 at 1 μM during transfection (Fig. S2E).

### 3.8 NFIB Is Required for BRN2 Induced Cellular Migration Through an EZH2 Mediated Pathway

**Fig. 5:**
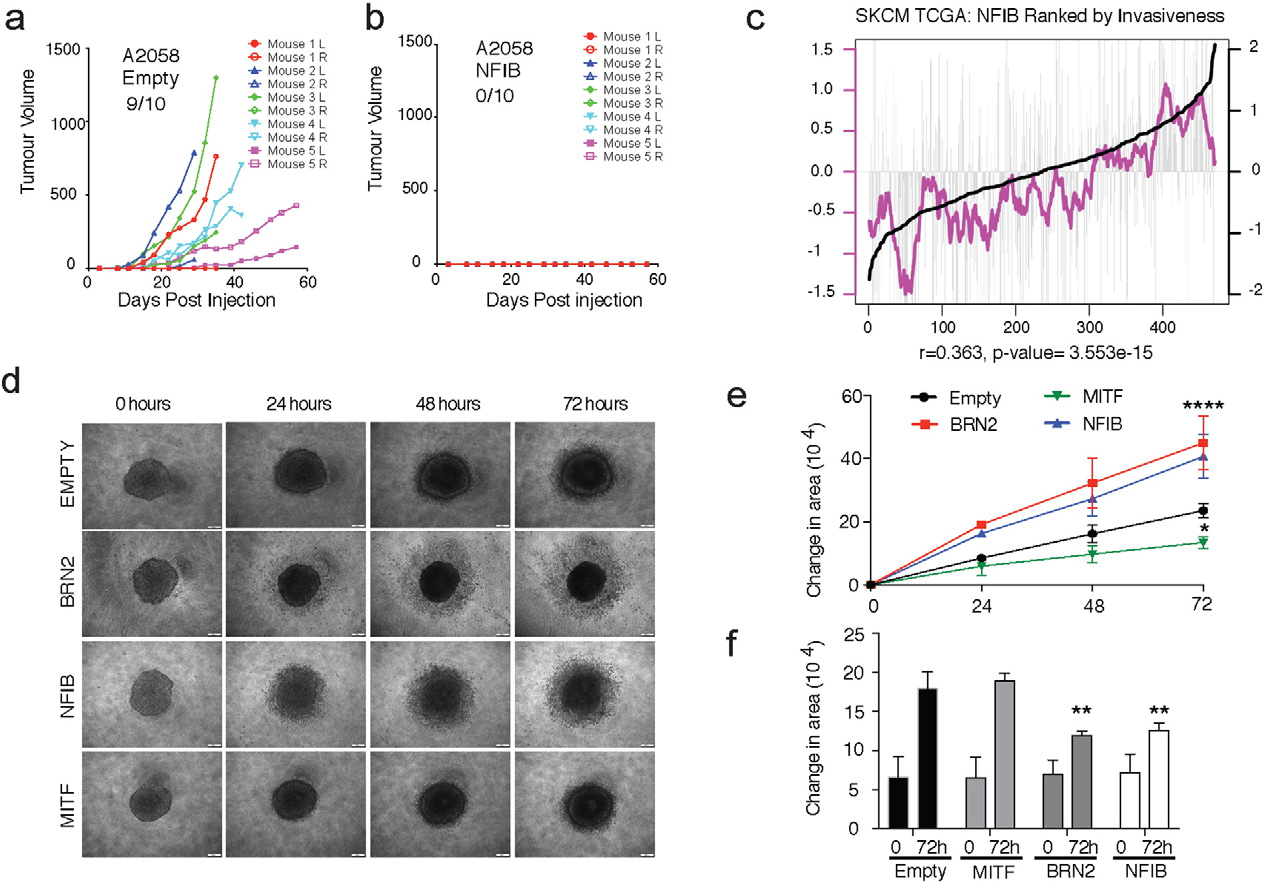
Overexpression of BRN2 decreases melanoma cell tumourigenicity but increases invasion. (A–B) 2 × 10^5^ A2058 human melanoma cells with stable over-expression of empty control or NFIB were injected subcutaneously into the hind flanks of five 5-week old male immunocompromised BALB/c *Foxn1*^*nu*^ mice. Three-dimensional measurement was performed two times per week, with tumour volume expressed as mm^3^. (C) Analysis of 471 melanoma samples in the TCGA dataset comparing NFIB expression and its correlation with a previously reported invasive gene signature (Verfaillie et al., 2015). Grey bars represent average expression of NFIB in each individual tumour, while the black line is a rank based on the average expression of the invasive gene signature used for initial sorting of the samples from low invasive to high invasive phenotypes. The purple line represents a moving average of NFIB expression per 20 tumours. Linear regression analysis reveals a Spearman *P*-value = 3.553e − 15 indicating a positive correlation between NFIB and invasiveness. (D) MM96L stable BRN2, MITF, NFIB, or empty human melanoma cells grown on agarose to generate 3D non-adherent melanoma spheroids. Spheroids were embedded in a collagen-media mixture and left to grow over a 72 h time-frame, with light phase photographs taken every 24 h. (E) Spheroid invasion was calculated from (D) by determining the change in the area of the invading cells disseminating away from the spheroid at 24 h time intervals relative to the 0 h timepoint. (F) The change in spheroid size was determined in (D) by measuring the change in area occupied by the spheroid alone (not the invasive populations) at 0 h vs. 72 h. *: *P* < 0.001, **: *P* < 0.001 ****: *P* < 0.0001. A two-way ANOVA with Tukey's post hoc test was performed on E–F. Changes in area occupied by invading cells and spheroid growth were calculated using ImageJ software. Data from (D–F) is representative of three independent experiments and is represented as the mean ± SEM. See also Fig. S3.

Our data suggests that NFIB functions as a downstream target of BRN2, which then potentially acts to increase EZH2 expression and decrease MITF expression. To determine if NFIB is required for the increased migration seen in melanoma cells over-expressing BRN2, A2058^− BRN2^, MM96L^− BRN2,^ and HT144^− BRN2^ cells were transfected with siRNA pools targeting NFIB or BRN2 itself (Figs. 4A, 5B, S2A, S2B and S2D). Wound healing assays were then performed on these cells and revealed, as expected, that siRNA mediated knock-down of BRN2 over-expression attenuated the elevated migratory capacity of these cells. Similarly, NFIB knockdown in these cells also resulted in a significantly reduced migration rate similar to levels seen in the BRN2 knockdown (Figs. 4A, 5B, S2A, S2B and S2D). Protein analysis of these cells revealed that NFIB is also required for BRN2 driven up-regulation of EZH2 expression, with EZH2 levels in A2058^− BRN2^ and MM96L^− BRN2^ cells falling to similar levels as that observed in the A2058^− empty^ control cells in response to both BRN2 and NFIB siRNA knock-down (Figs. 4C and S2C). This data suggests that NFIB is driving increased melanoma cell migration and increased expression of EZH2 downstream of BRN2.

EZH2 has previously been implicated in driving melanoma cell migration and invasion (Zingg et al., 2015). Accordingly the EZH2 inhibitor GSK343 was used to assess the contribution of EZH2 to NFIB driven melanoma cell migration in the A2058^− NFIB^ and MM96L^− NFIB^ cells. Stable cells were treated with 1 μM of EZH2 inhibitor and migration was assessed using wound-healing assays (Figs. 4D, 5E and S6B). Inhibition of EZH2 through GSK343 treatment resulted in a significant decrease in melanoma cell migration in the A2058^-empty^ MM96L^− empty^ control cells when compared with the vehicle (DMSO) control cells (Figs. 4D, 5E and S6B). Moreover, A2058^− NFIB^ and MM96L^− NFIB^ cells, previously shown to have elevated migration rates, were also found to have a significantly reduced migration in to the wound when treated with the inhibitor (Figs. 4D, 5E and S6B). Protein expression analysis was also performed and revealed that treatment with the inhibitor had little effect on NFIB or BRN2 expression in either cell line, however MITF expression increased in the A2058^− empty^ control cells in response to 1 μM GSK343 compared to the vehicle treatment (1.9 fold). Moreover, MITF levels that were previously decreased in A2058^− NFIB^ cells were restored following treatment with the EZH2 inhibitor (2.4 fold) (Fig. 5F).

### 3.9 MITF Re-expression Blocks NFIB Driven Increased Migration

Our data suggests that increased melanoma cell migration in response to elevated NFIB is in part achieved through EZH2 mediated down-regulation of MITF expression. This contention was further explored by restoring MITF expression in A2058^− NFIB^ and MM96L^− NFIB^ cells. The migratory capacity of both A2058/MM96L^− empty^ and A2058/MM96L^− NFIB^ cells, transduced 48 h previously with an empty (control) or MITF (MITF) expressing lentivirus, was determined (Figs. 4G, 5H and S6A). A2058^− empty^ and MM96L^− empty^ control cells were found to have a significantly decreased migration rate when MITF levels were increased by transient lentiviral treatment. Furthermore, the increased migratory capacity of A2058^− NFIB^ and M96L^− empty^ cells was significantly attenuated by lentiviral MITF expression and exhibited a migration rate similar to that of the empty control cell levels (Figs. 4G, 5H and S6A).

### 3.10 NFIB Overexpression Increases Invasion But Decreases Tumourigenicity

A rheostat model for MITF has been proposed that argues that the co-ordination of key tumourigenic activities such as initial tumour formation through to subsequent progression and metastatic dissemination is intimately linked to MITF expression levels (Carreira et al., 2006, Goding, 2011). To investigate the tumourigenic properties of NFIB expressing cell lines, A2058^− NFIB^ over-expressing human melanoma cells were injected into immune-compromised BALB/c *Foxn1*^*nu*^ mice in two different injection sites (left and right rear flank), and were culled once the ethical tumour burden was reached. Interestingly, while the A2058^− empty^ control cells were able to form tumours efficiently in 9/10 injection sites (Fig. 5A), A2058^− NFIB^ cells failed to form tumours (Fig. 5B). Protein analysis of these melanoma cell models prior to injection confirms that MITF has been drastically reduced in these NFIB lines (Fig. S3H).

In silico analysis of RNA-Seq data from 471 melanoma tumours contained in the TCGA database revealed a strong correlation between NFIB expression levels and an invasive gene signature (Fig. 5C). In this analysis, data was sorted and ranked from low to high expression of a previously reported invasive gene signature identified in melanoma tumours and cell lines (Verfaillie et al., 2015) with the line of best fit for this expression signature represented by the black line (Fig. 5C). Within this dataset, a grey bar represents level of NFIB expression for each individual tumour, and the purple line represents a moving average of NFIB expression per 20 melanomas across the sample set (Fig. 5C) with a strong association between NFIB levels and expression of the invasive gene set evident (Spearman correlation *P* = 3.553 × 10^− 15^).

**Fig. 6:**
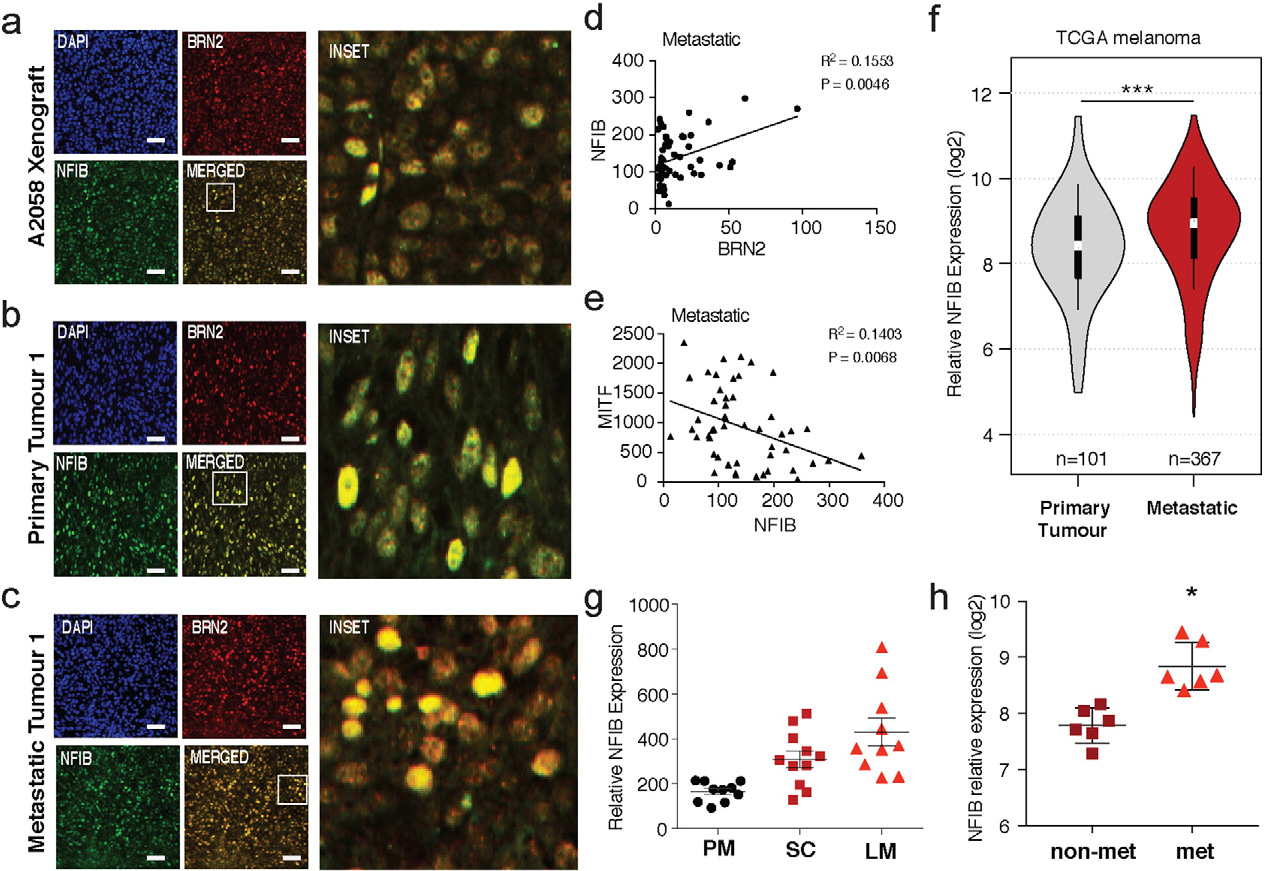
NFIB shows colocalisation with BRN2 in melanoma tumours and shows increased expression in aggressive/metastatic melanoma models. (A) Immunofluorescence microscopy on A2058 xenograft tumours surgically excised, formalin-fixed, and embedded in paraffin. Tumours were sectioned at 5 μm thickness and antigen-retrieved before labeling with BRN2 (red) and NFIB (green) antibody. DAPI was used to stain nuclei. (B-C) Immunofluorescence microscopy as described above on patient derived subcutaneous primary melanoma tumours and Lymph node metastatic melanoma tumours. (D) Microarray analysis of melanoma clinical samples representing 31 primary melanomas and 52 melanoma metastases from a previously published data set (Xu et al., 2008). Relative RNA expression was plotted and linear regression analysis was performed investigating the relationship between BRN2 and NFIB expression in metastatic samples. (E) Regression analysis on the above dataset looking at a correlation between MITF and NFIB expression in metastatic tumours. (F) Analysis of relative NFIB expression (log2 transformed) in 102 primary and 368 metastatic tumours from the TCGA dataset. Data represented as a violin plot and analyzed using the Mann-Whitney rank test. (G) Microarray analysis of subcutaneous tumours or lung metastases from immunodeficient mice injected subcutaneously or intravenously with a poorly-metastatic A375 melanoma cell line or with highly-metastatic derivative cell lines from a previously published dataset (Xu et al., 2008). Relative NFIB RNA expression was investigated in three specific groups; Poorly metastatic (PM), Subcutaneous tumours (SC), and the resultant lung metastases from the aforementioned subcutaneous tumours (LM). (H) Microarray analysis of relative NFIB expression (log2 transformed) in primary cutaneous melanomas derived from iMet (metastasis-capable) and iHRAS (non-metastatic) models from a previously published dataset (Scott et al., 2011). *: *P* < 0.05, ***: *P* < 0.001. A one-way ANOVA with a Tukey's post-hoc test was performed on (H). Data is represented as the mean ± SEM. Scale bars in white represent 200 μm. See also Figs. S3 and S6.

The effect of BRN2, MITF and NFIB over-expression in melanoma cell invasiveness was then determined using spheroids embedded in a collagen matrix, with invasion monitored at 24 h time points over a 72 h period (Fig. 5D). Quantification of cellular invasion into the collagen matrix revealed a significant increase in invasion of both BRN2 and NFIB over-expressing cell lines when compared with the empty vector control, whereas MITF over-expressing cells displayed a significantly impaired invasive capacity (Figs. 5D and 6E). A comparison of the overall growth of these spheres at 0 and 72 h also reveals that NFIB and BRN2 spheroid growth was significantly lower than the empty control and MITF spheroids, suggesting a decrease in their proliferation rate (Fig. 5F).

### 3.11 BRN2 and NFIB Show Co-localisation in Primary and Metastatic Human Tumours and NFIB Expression Is Correlated With Aggressive Melanoma

**Fig. 7:**
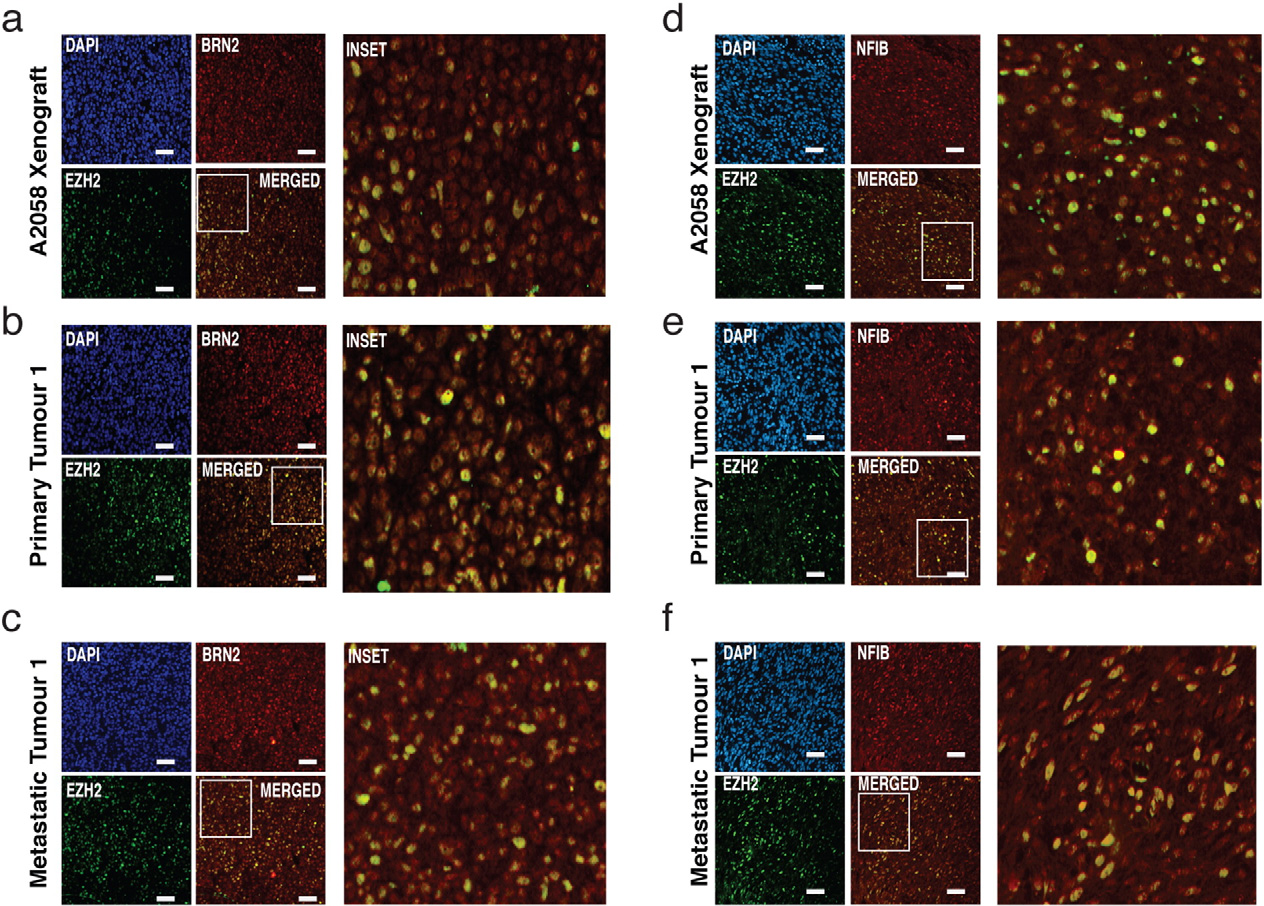
BRN2 and NFIB colocalise with EZH2 populations in vivo. (A) Immunofluorescence microscopy on A2058 xenograft tumours surgically excised, formalin-fixed, and embedded in paraffin. Tumours were sectioned at 5 μm thickness and antigen-retrieved before labeling with BRN2 (red) and EZH2 (green) antibody. DAPI was used to stain nuclei. (B–C) Immunofluorescence microscopy as described above on patient derived subcutaneous primary melanoma tumours and Lymph node metastatic melanoma tumours, labeled with BRN2 (red) and EZH2 (green). (D) Immunofluorescence microscopy on A2058 xenograft tumours as described above, labeled with NFIB (red) and EZH2 (green) antibody. (E–F) Immunofluorescence microscopy as described above on patient derived subcutaneous primary melanoma tumours and Lymph node metastatic melanoma tumours, labeled with NFIB (red) and EZH2 (green).

Previous studies have shown that BRN2 marks a distinct subpopulation of highly invasive cells located within melanoma tumours (Goodall et al., 2008, Pinner et al., 2009). Potential co-localisation of NFIB and BRN2 in vivo was initially examined in xenograft tumours generated in BALB/c *Foxn1*^*nu*^ mice using the A2058, MM96L, and HT144 human melanoma cell lines. Heterogeneous BRN2 and NFIB staining throughout the tumour was detected across all three tumour lines with co-localisation evident in most cells (Figs. 6A and S3D). Furthermore this co-localisation was also observed in human melanoma tissue excised from both primary and metastatic human tumours (Figs. 6B, 7C, S3E and S3f). Interestingly, there appeared to be a greater proportion of BRN2/NFIB positive cells in metastatic tumours when compared with the primary samples (not quantified). To further investigate changes in NFIB expression in human melanoma tumours, bio-informatic interrogation of a previously published gene expression micro-array data-set that analyzed RNA transcript levels of clinical melanoma samples representing 31 primary melanomas and 52 metastatic tumours (Xu et al., 2008) was performed. Linear regression analysis comparing the expression profiles of our various genes of interest reveals that there is a significant positive correlation between BRN2 and NFIB expression in human metastatic tumours (*P* < 0.0046; Fig. 6D). This analysis also revealed a significant inverse correlation between MITF and NFIB expression in metastatic tumour lines, with MITF levels decreasing as NFIB levels increase (*P* < 0.0068; Fig. 6E). Interestingly, both correlations were not significant when analyzed in the primary human tumour samples (Fig. S3B and S3C). This same study also employed a Patient Derived Xenograft (PDX) based strategy to identify highly metastatic tumour cells, which was then correlated with a metastatic gene expression signature using expression arrays (Xu et al., 2008). Tumours arising in the PDX model allowed stratification of tumours into non/poorly-metastatic (PM) and highly metastatic tumours with the latter group further sub-divided into samples taken from the sub-cutaneous tumour (SC) or a subsequent lung metastasis arising from the primary tumour (LM). Interrogation of the gene expression data set obtained from the PM, SC and LM tumour populations, reveals that relative NFIB expression is increased in the highly metastatic tumours, both subcutaneous (SC) and lung metastases (LM), when compared with the poorly metastatic (PM) tumour group (Fig. 6G). Interestingly, lung metastases also had greater relative NFIB expression when compared with the subcutaneous tumours, suggesting that NFIB expression is increased in more aggressive melanomas (Fig. 6G). Further interrogation of the TCGA melanoma database, investigating primary (101) vs. metastatic (367) derived patient tumours also reveals that relative NFIB expression is significantly increased in the metastatic tumour group (*P* < 0.001)(Fig. 6F) and that there is a significant correlation between BRN2 and NFIB expression within both groups (Fig. S6C). Finally, interrogation of an independent data set from an alternative xenograft study (Scott et al., 2011) also demonstrated elevated NFIB levels in highly metastatic tumours compared to the non-metastatic tumours (*P* < 0.05) (Fig. 6H).

### 3.12 BRN2 and NFIB Colocalise With EZH2 Populations In Vivo

We have been able to dissect a BRN2-NFIB-EZH2 axis that drives a highly invasive and migratory cell phenotype in vitro. To examine whether BRN2 and NFIB expression is linked with EZH2 expressing populations within tumours, colocalisation was examined in xenograft tumours generated in BALB/c *Foxn1*^*nu*^ mice as previously described using A2058 melanoma cells. EZH2 was expressed heterogeneously throughout each tumour, and showed distinct colocalisation with both BRN2 (Fig. 7A) and NFIB (Fig. 7D). Importantly, we find that the large majority of EZH2 negative populations are also BRN2 and NFIB negative. This colocalisation is also observed in primary human melanoma tumours (Fig. 7B, D) and in metastatic human tumour samples (Fig. 7C, E).

## 4 Discussion

Cellular heterogeneity has been linked to progression and therapeutic resistance in numerous cancer types (Marusyk et al., 2012). In melanoma, heterogeneous expression of the BRN2 and MITF transcription factors has been proposed to constitute a crucial switching mechanism between invasive and proliferative phenotypes (Goodall et al., 2008, Hoek et al., 2008, Hoek and Goding, 2010, Pinner et al., 2009, Quintana et al., 2010, Thurber et al., 2011). MITF has been identified as a crucial oncogene in melanoma, and accumulative evidence supports the hypothesis that its expression acts as a rheostat in controlling cell cycle behavior and migration/invasion (Carreira et al., 2006, Goding, 2011). In the context of phenotype switching, MITF expression has been characterized as a driver of a highly proliferative, less invasive cell state (Hoek et al., 2008, Vandamme and Berx, 2014). Conversely, BRN2 has been implicated in promoting an invasive and less differentiated state that is important in driving tumour progression towards metastasis, however few downstream targets of BRN2 that facilitate this phenotype have been identified (Arozarena et al., 2011, Boyle et al., 2011, Pinner et al., 2009). Moreover, the mechanism that underpins the switch between BRN2/MITF expression and associated phenotypes remains poorly understood. Here we report the regulation of expression of the NFIB transcription factor by BRN2 in melanoma cells, which in turn acts to increase cell migration and potentially invasion through the positive and negative regulation of the epigenetic regulator EZH2 and MITF respectively.

The expression and function of NFIB has not previously been explored in melanoma, but has been characterized as an oncogene in various cancers and is frequently amplified in epithelial type cancers (Chang et al., 2013, Dooley et al., 2011, Persson et al., 2009). Interestingly, NFIB activity in the skin has been demonstrated to be a key regulator of stem cell behavior in melanocytic stem cell niches (Chang et al., 2013). In this context, it has been shown that knockout of NFIB within this shared niche results in premature differentiation and proliferation of melanocytic stem cells. Similarly, BRN2 expression has been shown to maintain an undifferentiated melanoblast like cell phenotype during melanocyte development (Cook et al., 2003, Cook et al., 2005). Using a cultured human primary MB cell system, we observed high levels of BRN2 and NFIB protein in melanoblast cells that decreased dramatically over the course of differentiation into mature melanocytes. This role for BRN2 in facilitating a less differentiated phenotype has been observed in sub-sets of cells within melanoma tumours that are known to be more invasive (Pinner et al., 2009). The concept that cells undergo a process of de-differentiation to a more stem-cell like phenotype to help enhance metastasis has been well documented in various cancer models (Cheng et al., 1999, Friedmann-Morvinski and Verma, 2014, Sell, 1993). Immunostaining of NFIB within both mouse xenograft and human primary/metastatic melanoma tumours shows clear co-localization with BRN2 expressing populations, indicating that NFIB expression within melanoma tumours is most likely linked with these more undifferentiated and invasive cellular populations previously associated with BRN2 expression (Goodall et al., 2008, Pinner et al., 2009).

ChIP-ChIP experiments have detected BRN2 binding at the NFIB locus in vivo using 501 Mel human melanoma cells (Kobi et al., 2010). This observation, coupled with the reciprocal expression of NFIB in response to gain or loss of BRN2 in 2D culture and 3D melanoma spheres, and the co-expression seen in vivo suggests that there is likely a direct regulation of NFIB by BRN2 in melanoma cells. MITF knockdown within these models is shown to increase both NFIB and EZH2 expression. While there has been no clear direct interaction between MITF and these genes, previous studies have shown that knockdown of MITF leads to an increase in BRN2 expression (Thurber et al., 2011), suggesting that MITF may regulate NFIB and EZH2 indirectly through interactions with BRN2. The increases seen in melanoma spheroid invasion in both BRN2 and NFIB stable melanoma cells coupled with the loss of enhanced migratory capacity of BRN2 over-expressing cells when NFIB is knocked down suggests that this factor is a pivotal component of driving this highly migratory/invasive melanoma cell phenotype within BRN2 expressing populations. Crucially, recent reports from studies using rodent models of Small Cell Lung Cancer (SCLC) have suggested a vital role for NFIB in triggering invasive behavior that drives metastatic spread of these tumours (Denny et al., 2016, Semenova et al., 2016). In these models NFIB expression was found to be necessary and sufficient to support metastatic spread, an observation further supported by a correlation between high NFIB expression and an advance metastatic tumour grade in neuro-endocrine tumours from human patients (Semenova et al., 2016). Our data presented here would suggest NFIB is capable of propagating the acquisition of a more invasive phenotype through broad changes in chromatin status, in large part by increasing expression and function of the histone methyl-transferase enzyme EZH2. Consistent with this idea, the pivotal role NFIB plays in the acquisition of a metastatic phenotype in SCLC cells was identified in studies initially aimed at characterizing genome wide alterations in chromatin accessibility during metastatic progression of SCLC. The notable enrichment of NFIB binding sites in these hyper-accessible regions of the genome ultimately revealed the role for NFIB in driving metastasis and maintaining this permissive chromatin state (Denny et al., 2016).

Expression of the chromatin modifying enzyme EZH2 has been shown to be directly controlled by NFIB in neuronal stem cells (Piper et al., 2014). Interestingly, EZH2 has previously been characterized as a potent driver of melanoma metastasis with high levels associated with a poor patient prognosis (Bachmann et al., 2006, Fan et al., 2011, Luo et al., 2013, Zingg et al., 2015). Previous in silico screens searching for high-affinity NFI-binding sites within various polycomb repressive complex gene sets identified two putative, highly conserved NFI-binding motifs in the basal promoter of EZH2 which was confirmed by in vitro and ChIP analyses (Piper et al., 2014). We were able to demonstrate that EZH2 is up-regulated following both BRN2 and NFIB stable overexpression in melanoma cells. EZH2 up-regulation is lost in A2058^− BRN2^ and MM96L^− BRN2^ cells following NFIB siRNA knockdown, suggesting that NFIB expression is crucial to this regulation. Furthermore, mutating the NFI-binding sites in our EZH2-promoter driven luciferase construct effectively blocked the increases seen in EZH2-promoter driven expression. Interestingly, EZH2 expression has previous been linked with both increased growth and metastasis within tumours, whereas our model is predicated on a slowly proliferative cell phenotype. Unfortunately, NFIB cells failed to form tumours in xenograft models, which did not allow us to assess tumour growth. While melanoma cells often behave differently within the tumour microenvironment compared to cultured cells, it is possible that BRN2 and NFIB regulate additional genes that counteract the growth phenotype that might be anticipated with high EZH2 expression. Recent intravital imaging studies have found that EZH2 marks a heterogeneous, highly motile subpopulation of cells within melanoma tumours that is thought to promote early stages of metastasis (Manning et al., 2015). This study links these populations back to earlier intravital evidence showing that BRN2 expression is required for melanoma cell motility within tumours and dissemination into the blood stream during metastasis (Pinner et al., 2009). Our data provides strong evidence showing that these heterogeneous EZH2 expressing tumour populations are also BRN2 and NFIB positive and are likely mediated by BRN2 expression in an NFIB dependent manner. While our data demonstrates that NFIB directly promotes EZH2 expression downstream of BRN2, NFIB has been shown to repress EZH2 in primary cortical cells *in vitro* and during cortical development in-vivo (Piper et al., 2014) suggesting that cellular specificity and importantly cellular context may determine if NFIB functions as a repressor or activator at this locus. Computational predictions, coupled with multiplex experimental analysis suggest that in most contexts, the NFI family of transcription factors primarily act as transcriptional activators (Pjanic et al., 2011).

MITF has previously been described as having both oncogenic and tumour suppressor properties within melanoma (Hartman and Czyz, 2015, Levy et al., 2006). This idea is further perpetuated by the fact that while BRN2 and MITF have been shown to be expressed in two mutually exclusive populations, BRN2 is able to both directly increase and decrease MITF expression in a context specific manner (Goodall et al., 2008, Thurber et al., 2011, Wellbrock et al., 2008). The phenotype-switching model of tumour progression dictates that these two mutually exclusive populations are able to selectively switch back and forth to drive tumour growth through a predominately proliferative cellular phenotype driven by MITF, and tumour metastasis by an invasive BRN2 directed cellular population (Hoek et al., 2008, Hoek and Goding, 2010). Evidence is emerging to suggest that this model is more fluid than previously thought, and that cells with considerably lower invasive capacity are able to contribute to metastatic populations through a cooperative invasion mechanism with highly invasive cells, dependent on protease activity and fibronectin deposition (Chapman et al., 2014, Haass et al., 2014). Despite such other models emerging, the link between BRN2 expression and invasiveness is well established (Arozarena et al., 2011, Boyle et al., 2011, Kobi et al., 2010) which may be achieved in large part due to the impact that BRN2 activity may have on the MITF rheostat model, whereby invasion is driven by low levels of MITF and proliferation is driven at higher levels (Carreira et al., 2006). NFIB in this context would appear to function downstream of BRN2 and is shown to repress MITF expression, likely through the up-regulation of EZH2 expression and activity. The tumourigenic assays performed here show that NFIB overexpression impaired the ability of these cells to form tumours in nude mice at the site of injection. Analysis of protein expression in these injected NFIB lines reveals that MITF levels are effectively silenced. In this context, stable over-expression of NFIB limits the ability of these cells to upregulate vital components such as MITF, that may be required for the cells to adapt to changes in the local microenvironment in order to establish tumours. Analysis of both proliferation and invasion within our spheroid assays clearly show that NFIB drives a slow cycling, highly invasive cellular phenotype. While such a phenotype would likely drive a highly metastatic cell state, it may not allow tumour formation at both primary and distal sites. Thus, our results suggest that MITF levels regulated by NFIB downstream of BRN2 are key in tumour formation and growth. Importantly, we are also able to show that NFIB likely regulates MITF by increasing the levels of EZH2. As EZH2 acts to repress transcription via chromatin methylation, such a mechanism suggests that NFIB is able to regulate MITF in a way that is both dynamic and reversible, which would be a key feature for switching between cell states that drives growth versus metastasis. Specifically, NFIB expression and regulation within tumours may be key in feeding into the MITF rheostat model downstream of BRN2, whereby changes in BRN2 are able to dictate reversible changes in MITF expression through NFIB and EZH2 to a level that will allow for increased proliferation during formation, and increased migration/invasion when undergoing a switch to a more EMT-like invasive phenotype during metastasis. Moreover, our analysis of NFIB function in melanoma cells, together with recent evidence of NFIB as a powerful driver of SCLC metastasis suggest that NFIB may play a broader role in metastatic spread of other cancers. Importantly, these studies reveal that NFIB has the ability to promote dynamic changes in the chromatin state of tumour cells to facilitate migration, invasion, and metastasis. While our study reveals the regulation of the EZH2 chromatin modifying enzyme by NFIB is a key conduit of this effect in melanoma cells, it remains to be determined if a similar epigenetic axis is governed via an NFIB-EZH2 axis in other tumour types to drive invasion and metastasis.

## Appendix A Supplementary data

Supplementary material Image 1
