# Supplementary material for: NFIB Mediates BRN2 Driven Melanoma Cell Migration and Invasion Through Regulation of EZH2 and MITF
Source: eBioMedicine. 2017 Jan 16;16:63–75. doi: 10.1016/j.ebiom.2017.01.013 (PMC5474438; doi:10.1016/j.ebiom.2017.01.013)
Supplement: Supplementary file 1 — Supplementary material [file mmc1.docx]

**Supplemental Information**

Figure S1. Related to Figure 4. NFIB drives a highly migratory phenotype across multiple cell lines

**
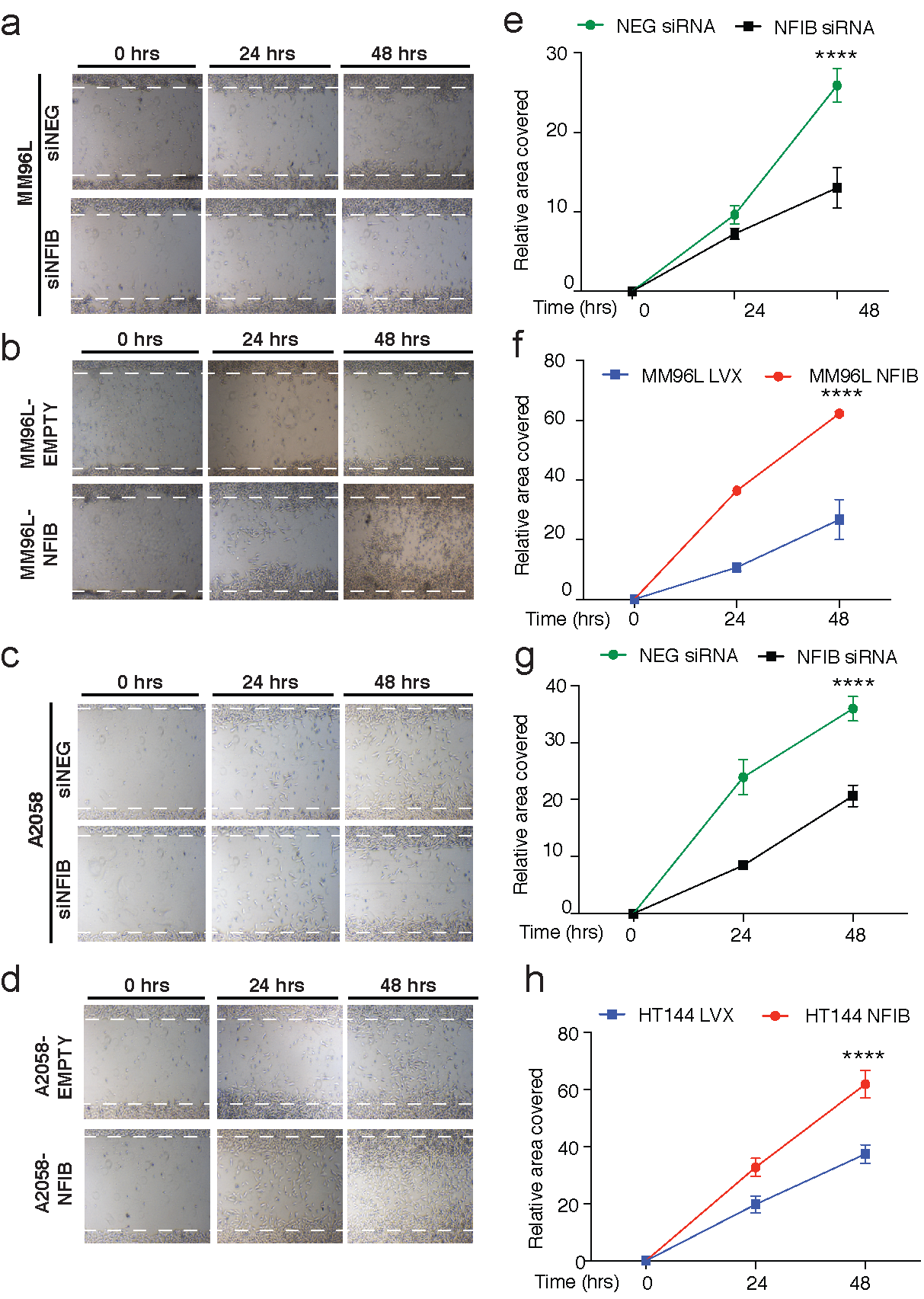
**

Figure S2. Related to Figures 4 and 5. NFIB drives BRN2 mediated migration potentially via the knockdown of MITF

**
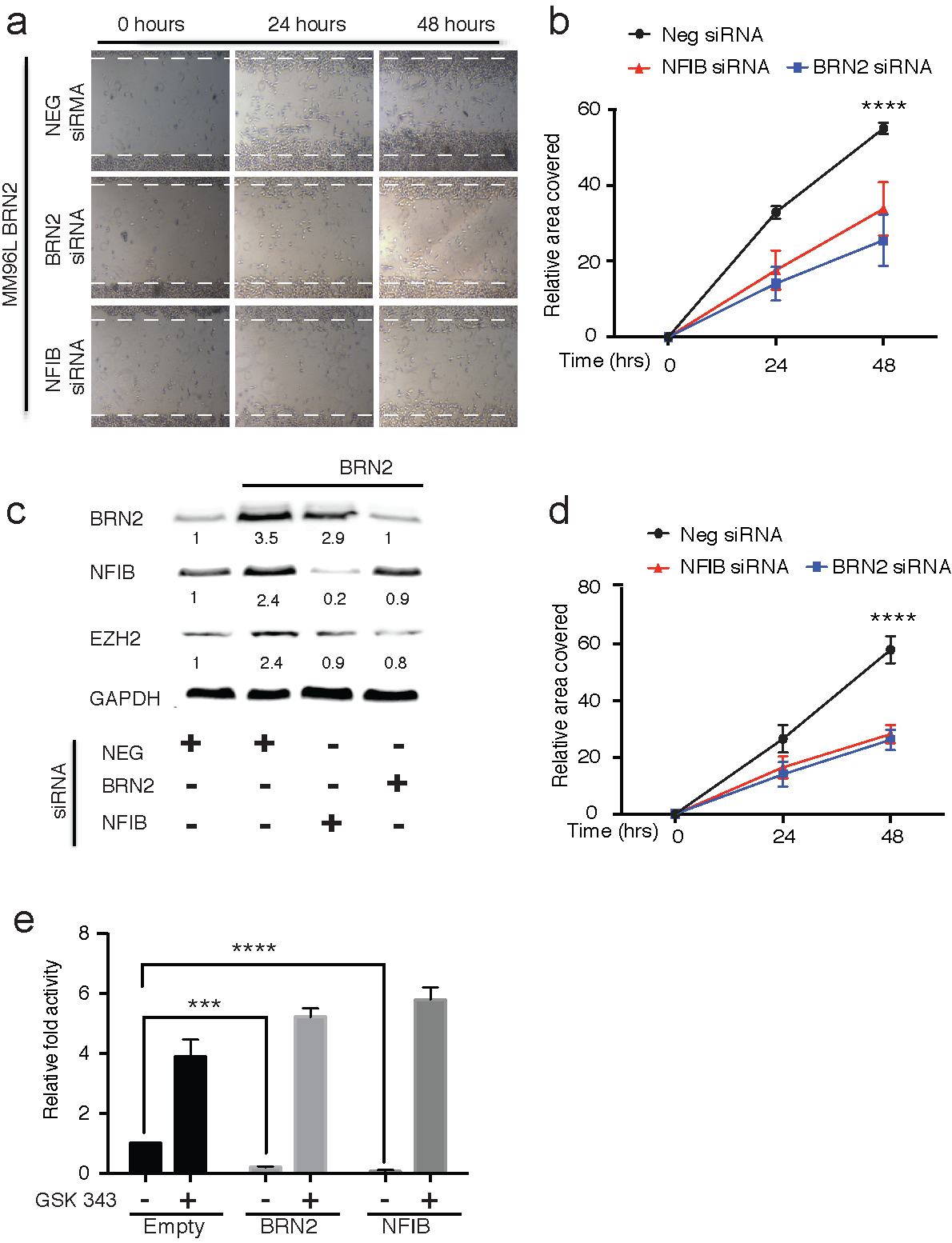
**

Figure S3. Related to Figures 1, 4, 6 and 7. NFIB localizes with BRN2 in development and within *in vivo* models

**
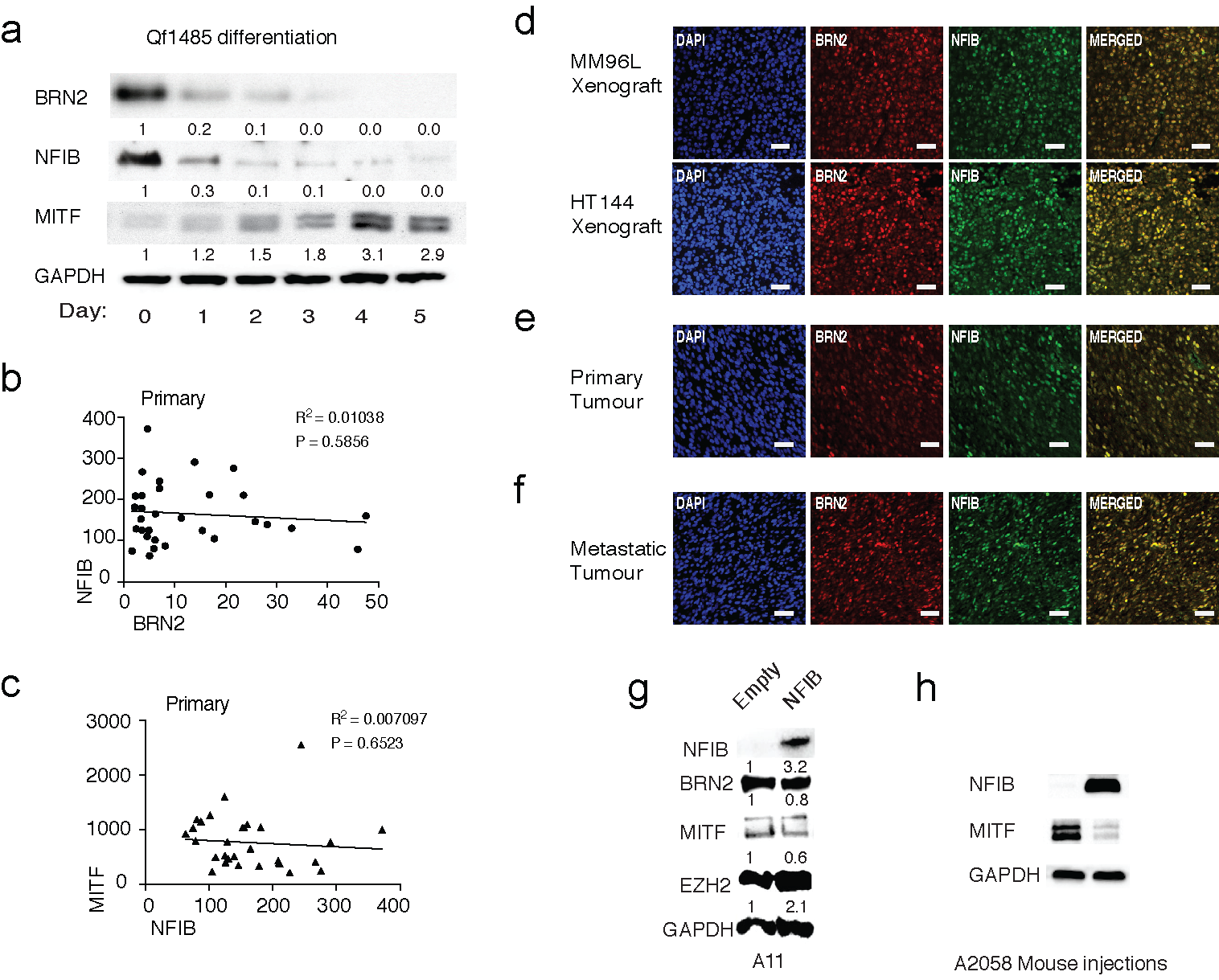
**

Figure S4. Related to Figure 3. MITF negatively regulates EZH2 and NFIB expression

**
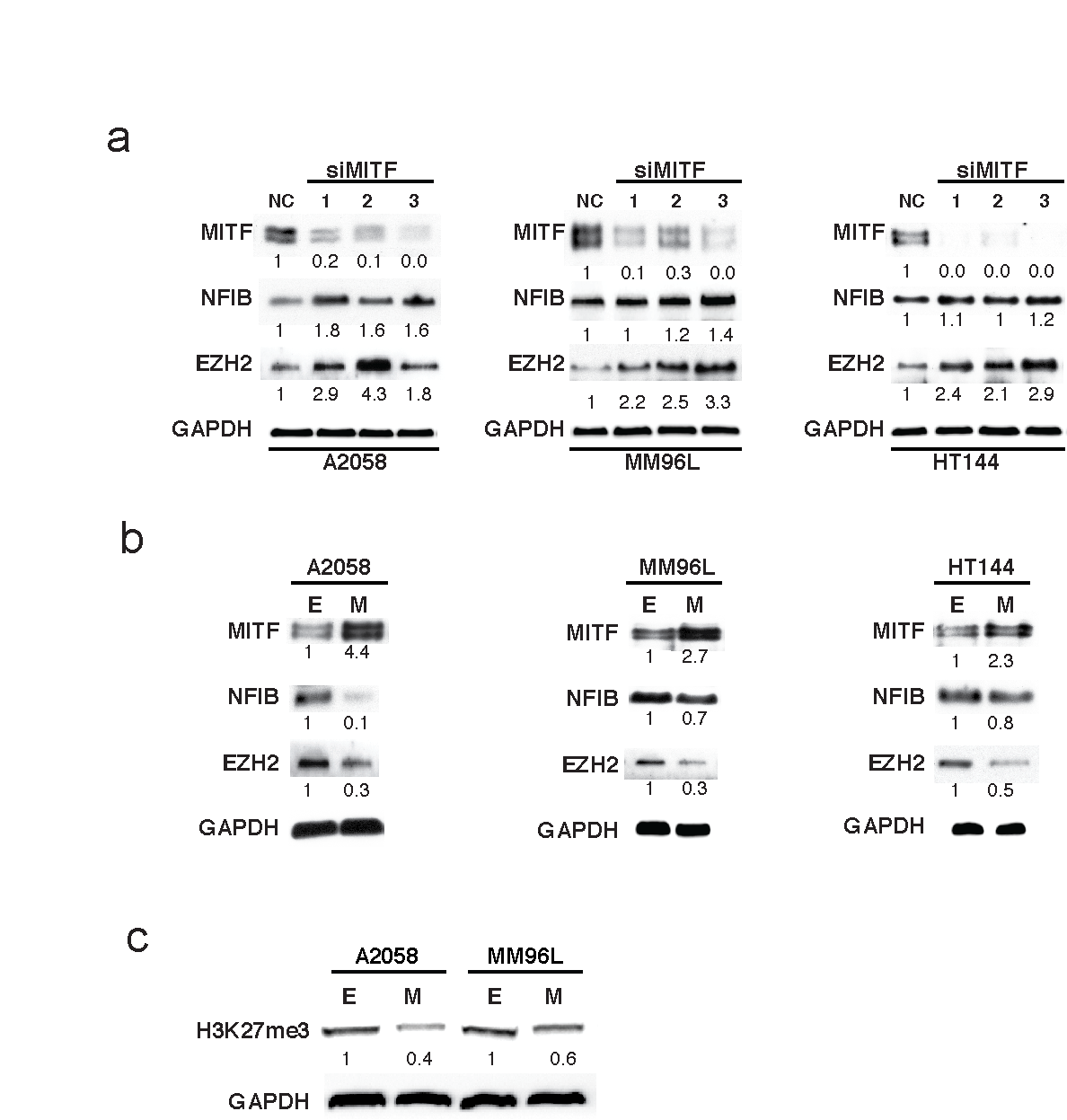
**

Figure S5. Related to Figure 4. Investigating migratory potential in melanoma cells following BRN2/MITF manipulation


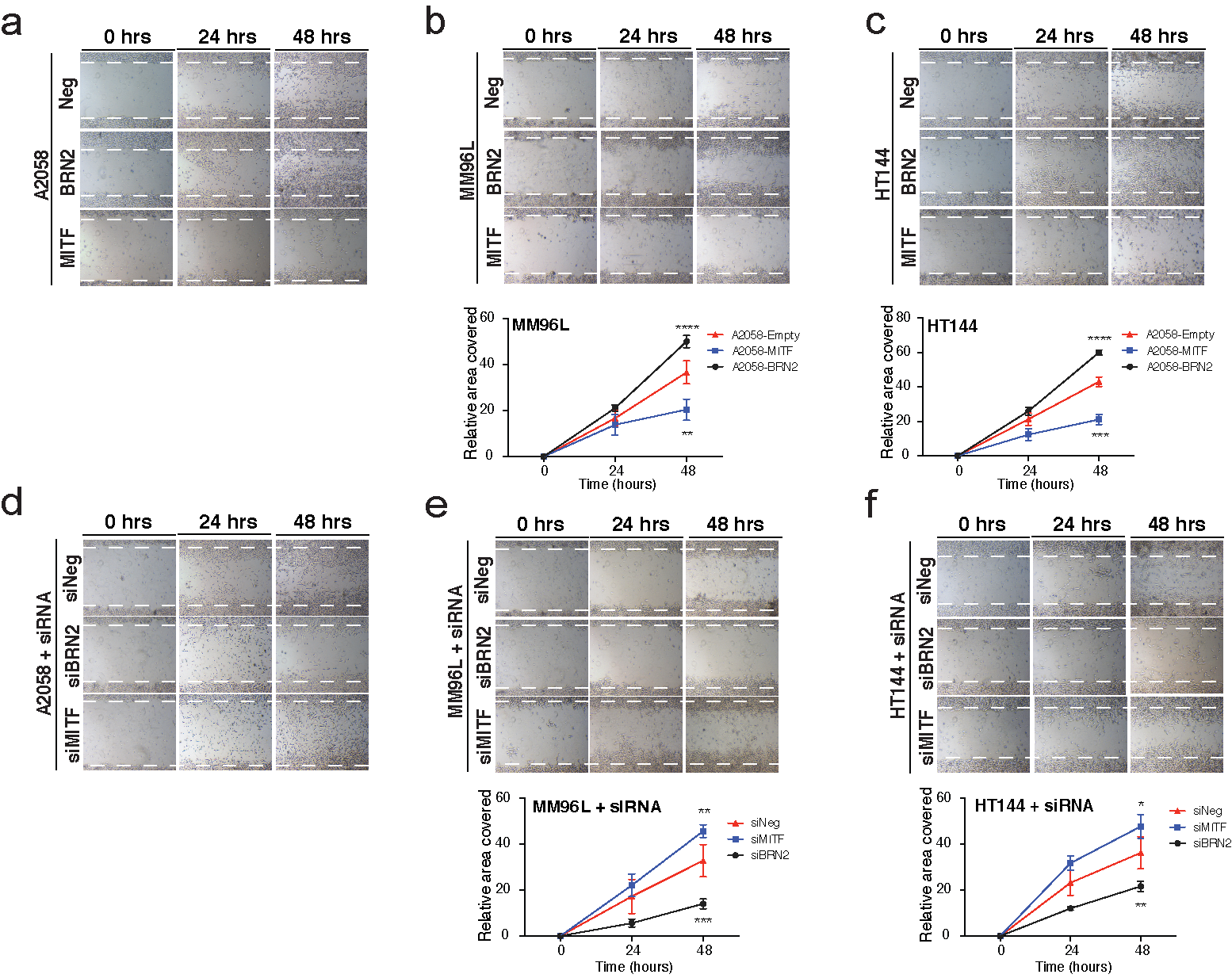


Figure S6 Related to Figures 5 and 7. NFIB drives migration of BRN2 through interactions with EZH2 and MITF

**
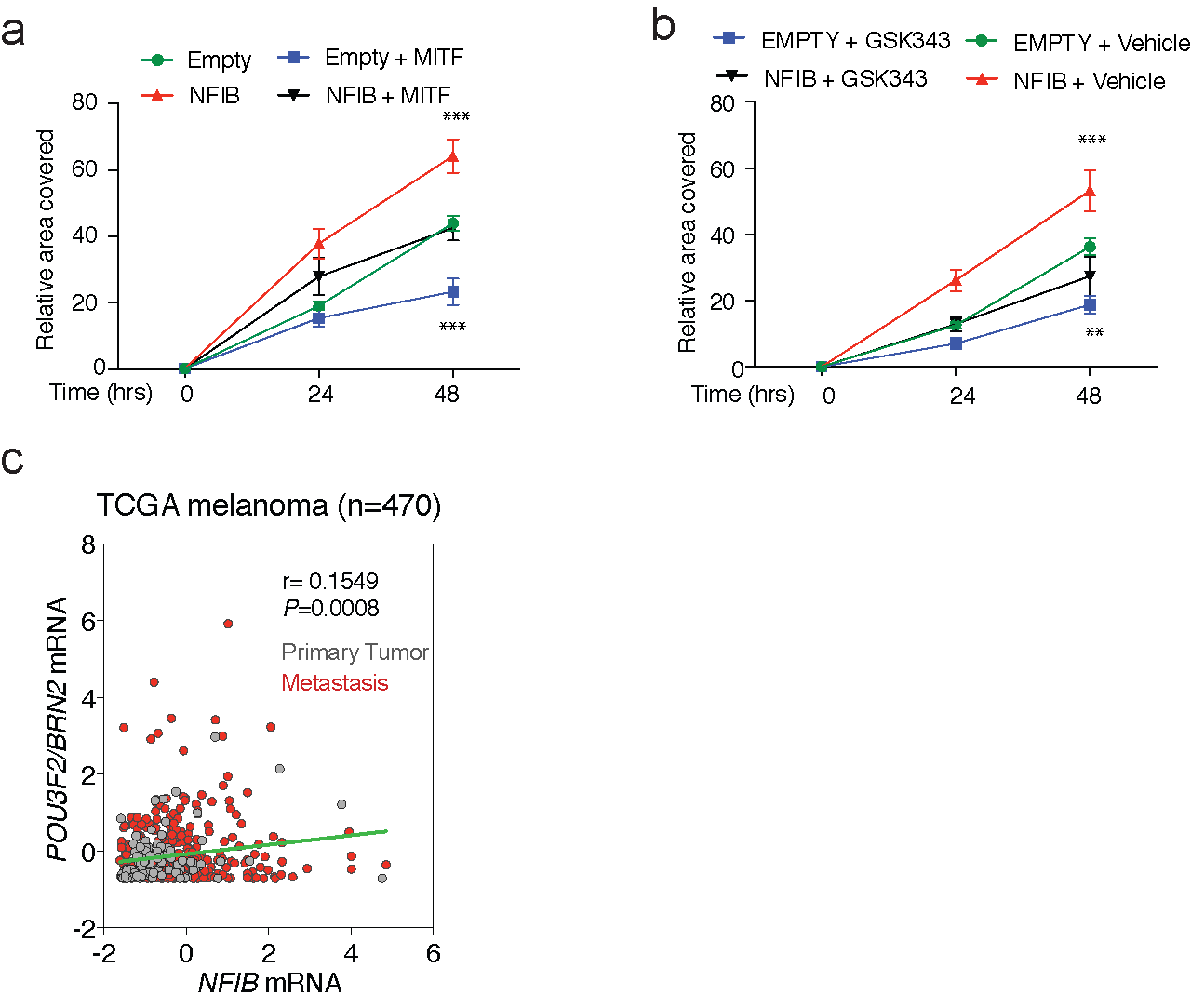
**

Figure S7 Related to Figures 2 and 3. BRN2 regulates NFIB expression in 3D melanoma spheres

**
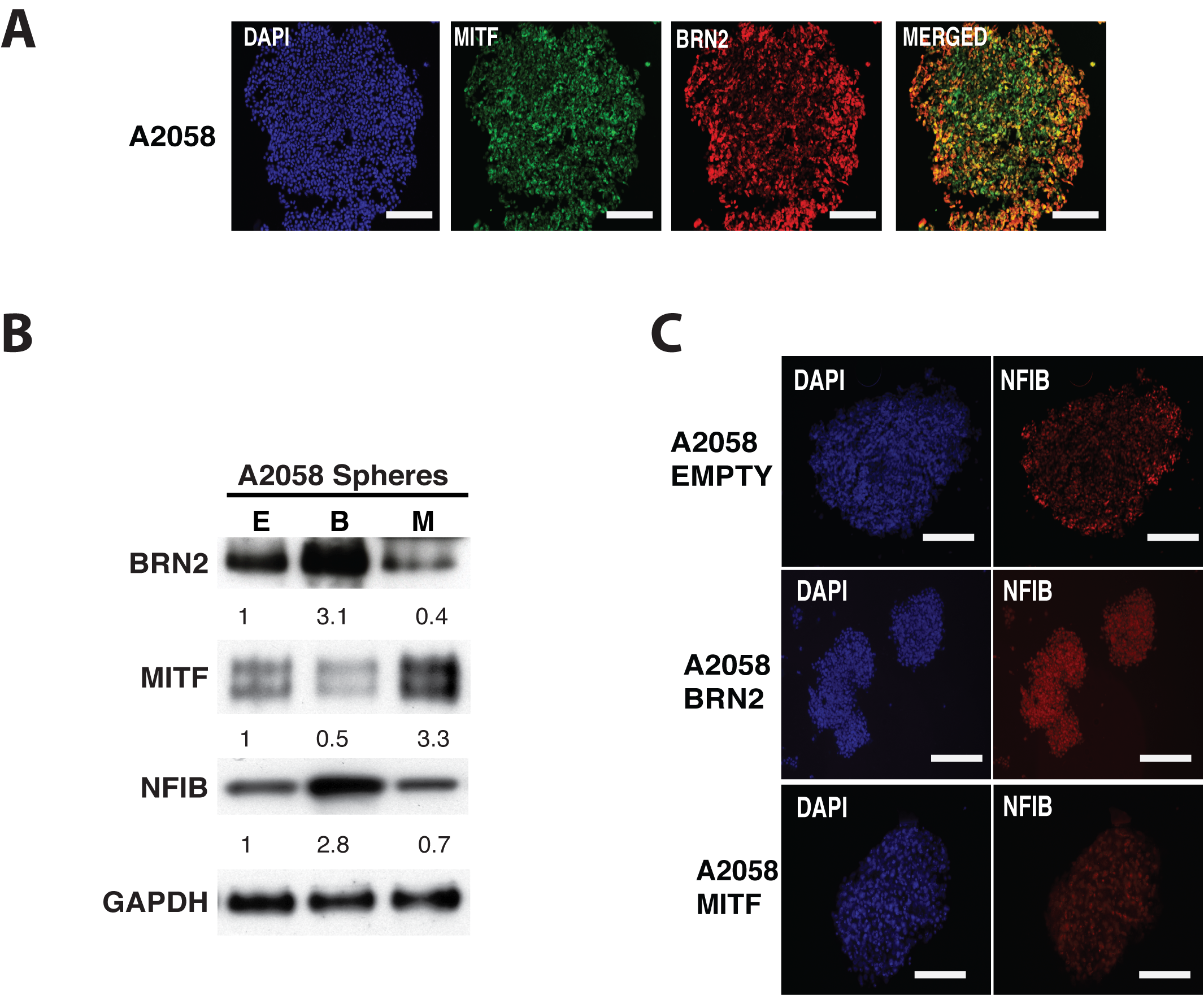
**

**Figure S1. Related to Figure 4. NFIB drives a highly migratory phenotype across multiple cell lines**

(A) Wound healing assays were performed in MM96L human melanoma cells treated with siRNA against NFIB or a scrambled control 24 hours prior to commencement of the experiment. Light-phase photographs were taken of wounds at identical points immediately (0 h) and 24 h and 48 h post-wounding. Representative images taken from three independent experiments are shown, and (E) were quantified measuring the area covered at each time point relative to the initial 0 hour time point using the TScratch software. (B) Identical wound healing assays were preformed in MM96L NFIB stable melanoma cells and empty control cells (F) and were quantified as described above. ****:P<0.0001. (C) Light-phase images at 0, 24 and 48 hours of wound healing assays performed in A2058 human melanoma cells treated with siRNA against NFIB or a scrambled control 24 hours prior to commencement of the experiment (quantifications found in Figure 4G) (D) Light-phase images at 0, 24 and 48 hours of wound healing assays performed in A2058 NFIB stable melanoma cells (quantifications found in Figure 4H)(G) Wound healing assays were performed in HT144 human melanoma cells treated with siRNA against NFIB and were quantified as mentioned above. (H) Identical wound healing assays were performed in HT144 NFIB stable melanoma cells and quantified as above. Data is represented as the mean ± SEM and analysed using a two-way ANOVA with Tukey’s pos hoc test.

**Figure S2. Related to Figures 4 and 5. NFIB drives BRN2 mediated migration potentially via the knockdown of MITF**

(A) Wound healing assays were performed in BRN2 stable MM96L human melanoma cells treated with siRNA against NFIB, BRN2 or a scrambled control 24 hours prior to commencement of the experiment. Light-phase photographs were taken of wounds at identical points immediately (0 h) and 24 h and 48 h post-wounding. Representative images taken from three independent experiments are shown, and (B) were quantified measuring the area covered relative to the initial 0 h scratch at each time point using the TScratch software. ****:P<0.0001 compared with the scrambled negative control. (C) Immunoblot analysis investigating protein expression was performed on the cells described above, along with an MM96L Empty control. Antibodies were used against BRN2, NFIB, and EZH2 as labeled. GAPDH served as the loading control, with the normalized ratio of expression (Empty set to 1) indicated below each image. Data representative of three separate experiments (D) Wound healing assays were performed as described above in HT144 BRN2 stable cells treated with siRNA against NFIB, BRN2 or a scrambled control 24 hours prior to commencement of the experiment and were quantified as described. ****:P<0.0001 comparing NFIB/BRN2 siRNA with the negative scrambled control. (E) A2058 stable BRN2, NFIB, and empty control melanoma cells were transfected with a reporter construct containing the MITF immediate promoter region driving luciferase expression and subsequently treated with GSK343 or vehicle control (DMSO) at 1 μM. Relative luciferase expression was measured and normalized against the empty DMSO control to represent relative fold activity. ***:P<0.001, ****:P <0.0001. Data is represented as the mean ± SEM and was analysed using a two-way ANOVA with Tukey’s post hoc test.

**Figure S3. Related to Figures 1, 4, 6 and 7. NFIB localizes with BRN2 in development and within *in vivo* models**

(A) Immunoblot analysis on QF1485 primary human melanoblast cells induced to differentiate into pigmented melanocytic like cells over a 5-day period,. Whole cell lysates taken at each day of differentiation were analyzed for BRN2, NFIB and MITF levels as indicated. GAPDH served as the loading control and the normalized ratio of each extract (day 0 set to 1) was indicated below each image. (B) Microarray analysis of melanoma clinical samples representing 31 primary melanomas and 52 melanoma metastases from a previously published dataset ([Xu et al., 2008](#_ENREF_40)). Relative RNA expression was plotted and linear regression analysis was performed investigating the relationship between BRN2 and NFIB expression in primary melanoma samples. A significant correlation was deternined at P<0.05. (C) Regression analysis on the above dataset looking at a correlation between MITF and NFIB expression in primary tumours. (D-E) Immunofluorescence microscopy of MM96L and HT144 xenograft tumours as labeled that were surgically extracted, formalin fixed, and embedded in paraffin. Tumours were sectioned at 5μm thickness and antigen-retrieved before labeling with BRN2 (red) and NFIB (green) antibody. DAPI was used to stain nuclei and scale bars represent 200μm. (E-F) Immunofluorescence microscopy as described above in subcutaneous primary melanoma tumours and Lymph node metastatic melanoma tumours from patient samples. (G) Relatively low NFIB expressing A11 human melanoma cells were subjected to lentiviral transduction to induce stable over-expression of NFIB and were immunoblotted with antibodies against BRN2, MITF, NFIB, EZH2, and GAPDH. The GAPDH normalized ratio of expression (E set to 1) was indicated below each image. (H) Immunoblot analysis of the A2058 human melanoma stable NFIB and empty control cells used for xenograft studies indicating NFIB and MITF protein expression. GAPDH was used as a loading control.

**Figure S4. Related to Figure 3. MITF negatively regulates EZH2 and NFIB expression**

(A) Immunoblot analysis on A2058, MM96L, and HT144 human melanoma cells treated with three different siRNA directed against MITF, or a negative siRNA control. GAPDH served as the loading control, with the normalized ratio of expression (NC set to 1) indicated below each image. (B) Melanoma cell lines subjected to lentiviral transduction to induce stable over-expression of MITF immunoblotted with antibodies, with GAPDH used as a loading control. The normalized ratio of expression (E set to 1) was indicated below each image. (C) Stable MITF overexpressing A2058 and MM96L protein lysates from above underwent further immunoblot analysis to investigate H3K27 tri methylation status. GAPDH was used as a loading control, with the normalized ratio of expression (E set to 1) indicated below each image. Data representative of three independent experiments.

**Figure S5. Related to Figure 4. Investigating migratory potential in melanoma cells following BRN2/MITF manipulation**

Wound healing assays were performed in (A) A2058 (B) MM96L and (C) HT144 human melanoma cells with stable overexpression of BRN2, MITF, and an empty vector control. Light-phase photographs were taken of wounds at identical points immediately (0 h) and 24 h and 48 h post-wounding. Representative images taken from three independent experiments are shown, and the area covered at each time point relative to the initial 0 hour time point were quantified below the images using TScratch software (Quantifications for A are in Figure 4F). Identical experiments were performed across (E) A2058, (F) MM96L, and (G) HT144 melanoma cells treated with siRNA specific to BRN2, MITF, and a scrambled negative control 24 hours prior to the commencement of the assay (Quantifications for D are in Figure 4E). *:P<0.05, **:P<0.01, ***:P<0.001, ****:P<0.0001. All stats performed were compared to either empty control or negative siRNA transfected cells. All data representative of three independent experiments.

**Figure S6 Related to Figures 5 and 7. NFIB drives migration of BRN2 through interactions with EZH2 and MITF**

(A) Wound healing assays were performed as described above in MM96L Empty and NFIB stable cells treated with MITF or empty control lentivirus 48 hours prior to commencement and were quantified as described above. ***:P<0.001 and compares Empty + MITF with Empty + control. ***:P<0.001 and compares NFIB + MITF with NFIB + control. (B) Wound healing assays were performed as described above in MM96L stable NFIB and Empty melanoma cells treated with GSK343 or a vehicle control (DMSO) at 1 μM and were quantified as described. **:P<0.01 and compares NFIB + Vehicle with NFIB + GSK343. ***:P<0.001 and compares EMPTY + Vehicle with EMPTY + GSK 343. (C) Linear regression analysis performed on 471 melanoma samples in the TCGA data set comparing relative (Log2) NFIB and BRN2 expression. Metastatic samples are represented in red while primary tumours are represented in gray.

**Figure S7 Figure 3 BRN2 regulates NFIB expression in 3D melanoma sphere model** (A) Immunofluorescence microscopy of BRN2 (red) and MITF (green) co-stained melanoma spheres generated from empty control A2058 human melanoma cells. DAPI was used to stain the nuclei.(B) Whole sphere lysates generated from empty, BRN2, and MITF A2058 melanoma spheres were analyzed by western blot with antibodies against BRN2, NFIB, MITF, and GAPDH(C) Immunofluorescence microscopy of NFIB (red) in melanoma spheres generated from empty, BRN2, and MITF stable A2058 melanoma cells. DAPI was used to stain the nuclei. Scale bars in white represent 200μm. All data representative of three independent experiments. Band expression intensity of western blots was normalized to the first lane (GAPDH used as a loading control) using ImageJ software and indicated below each blot.

**Supplemental experimental procedures**

**Lentivirus transduction**

BRN2 was cloned into pLVX and pTight lentiviral vectors and MITF and NFIB were cloned into pLVX alone (Clonetech). pTetoff advanced (Clonetech) was also cloned into pLVX according to the manufacturer's protocol and was co-transduced with all pTight lentiviral vectors. Lentivirus was then generated using the Lenti-X HT packaging system (Clontech) according to the manufacturer's protocol and melanoma cells were transduced as described previously ([Jagirdar et al., 2013](#_ENREF_2)).

**Immunofluorescence**

Fluorescent imaging of cells and melanoma spheres were performed as described previously ([Jagirdar et al., 2013](#_ENREF_2); [Thurber et al., 2011](#_ENREF_7)) Briefly, melanoma spheres were fixed in 4% PFA and embedded in 1.5% noble agar, sectioned using a Leica VT1000 S Vibratome at 50μm, and antigen-retrieved before labeling with antibody. The antibodies used for imaging were rabbit monoclonal BRN2 antibody ([Smith et al., 1998](#_ENREF_5)), rabbit polyclonal NFIB antibody (Sigma Aldrich; HPA003956), mouse monoclonal MITF antibody- C5/D5 (Sigma-Aldrich; 284M-9), mouse monoclonal EZH2 (Active Motif #39639), with DAPI used to stain nuclei. Melanoma biopsies were staged according to the 2002 American Joint Committee on Cancer staging system. Human primary tumours, lymph node metastatic tumours, and mouse xenograft tumours were formalin-fixed, embedded in paraffin and sectioned at a 5µm thickness. Tumours were dual stained using the TSA Plus Cyan 3 kit (Perkin and Elmer) as per the manufacturer’s protocol. Rabbit monoclonal BRN2 antibody ([Smith et al., 1998](#_ENREF_5)) was used at a dilution of 1:20000 and amplified using TSA, while rabbit polyclonal NFIB was used at 1:200 (Sigma Aldrich).

**Luciferase reporter assays**

The constructs and methods used in the luciferase assay for the EZH2 experiments were as previously described ([Piper et al., 2014](#_ENREF_3)). The construct used for the luciferase assay for the MITF experiments contained a 1.8Kb region of the MITF immediate promoter cloned into the PGLII basic promoter (Promega). DNA was transfected into A2058 cells treated with 1μM of the EZH2 inhibitor GSK343 or DMSO control using Lipofectamine 2000 (ThermoFisher) and was normalized to a PGLII basic empty control (Promega). 48 hours after transfection, luciferase activity was measured using the steadylite system (PerkinElmer) and was normalized to measure fold change activity.

**Quantitative Real-time PCR**

A2058 stably over-expressing BRN2, MITF, and empty control human melanoma cells underwent phenol-chloroform mediated RNA extraction following trizol (Sigma Aldrich) addition and reverse transcription was performed on 1μg RNA using iSCRIPT RT Supermix (Bio-rad) and subjected to QPCR analysis using SYBR Green mix on ViiA7 real time cycler (ABI). Real time primers used in the study were B2M (Fwd 5’CATTCGG5’GCCGAGATGTCT, Rev 5’CTCCAGGCCAGAAAGAGAGAGTAG), NFIA (Fwd 5’TCAATCTGAAAGTCCCAGCC, Rev 3’TGCAGCTATTGGTGTCTGTG), NFIB (Fwd 5’CTCTGCATCTCCACAGGATTC, Rev 3’GATAGCTTGTGTTGGAAATGGC), NFIC (Fwd 5’CAAGTCACCATTCAACAGCC, Rev 3’TCGTAGGGAAATGCAGAGC), and NFIX (Fwd 5’AGGACTGTTTTGTGACTTCCG, Rev 3’GGTTGATGTTGTAGTAGCTGGG). All samples were tested in triplicate and the housekeeping gene Beta/2-microglobulin was used to calculate relative changes in gene expression levels, which was then normalized to the fold change relative to the empty (control) cells.

**Wound healing migration assay**

A2058, MM96L, and HT144 human melanoma cell lines and stable cells were seeded in a 6-well plate and scratches were performed as previously described ([Smith et al., 2011](#_ENREF_6)). TScratch software was used to calculate the area covered by the cells at each given time point ([Geback et al., 2009](#_ENREF_1)).

**Bio-informatics analysis**

RNA-Seq data was downloaded from the TCGA database using cBioportal ([http://www.cbioportal.org](http://www.cbioportal.org/)). Individual gene expression values for genes of interest were retrieved as normalized RNA-Seq by Expectation Maximization (RSEM) read counts processed through the TCGA/cBioportal. To avoid negative expression values on log2-tranformation RSEM-values <1 were set to 1. Melanoma samples were ordered by increasing expression values of the averaged invasion signature ([Verfaillie et al., 2015](#_ENREF_8)). The moving average NFIB was calculated using a sample window size of n=20 and trendlines were added to the bar plots. A R-skript for calculating and generating moving average plots of TCGA cancer cohorts implementing TCGA access via cBioportal was provided previously ([Riesenberg et al., 2015](#_ENREF_4)). Relative NFIB expression of TCGA primary vs. metastatic melanoma samples was also investigated using a violin plot and analyzed using a Mann-Whitney rank test. All microarray expression analysis data was downloaded from the NCBI GEO DataSets database (<http://www.ncbi.nlm.nih.gov/gds>). Linear regression analysis was performed on microarray data from 31 primary and 52 metastatic human patient samples with NFIB expression plotted against BRN2, and MITF plotted against NFIB ([Xu et al., 2008](#_ENREF_40)). Array profiling was also performed on another dataset taken from this study in which samples were collected from immunodeficient mice that were injected subcutaneously with poorly metastatic human melanoma cell lines and highly metastatic cell lines. The highly metastatic lines had samples taken from the resulting subcutaneous tumours and the lung metastases ([Xu et al., 2008](#_ENREF_40)). Individual data points for relative NFIB expression were plotted and the mean $\pm$ SEM were presented as a scatter plot. Microarray analysis was performed and plotted as described previously from a simlar study investigating relative NFIB expression from samples that were collected from primary cutaneous melanoms derived from iMet (highly metastatic) and iHRAS (non-metastatic) models ([Scott et al., 2011](#_ENREF_37)). Data was analyzed using a one-way ANOVA with a Tukey’s post hoc test.

**Supplemental References**

Geback, T., Schulz, M.M., Koumoutsakos, P., and Detmar, M. (2009). TScratch: a novel and simple software tool for automated analysis of monolayer wound healing assays. Biotechniques *46*, 265-274.

Jagirdar, K., Yin, K., Harrison, M., Lim, W., Muscat, G.E.O., Sturm, R.A., and Smith, A.G. (2013). The NR4A2 Nuclear Receptor Is Recruited to Novel Nuclear Foci in Response to UV Irradiation and Participates in Nucleotide Excision Repair. Plos One *8*.

Piper, M., Barry, G., Harvey, T.J., McLeay, R., Smith, A.G., Harris, L., Mason, S., Stringer, B.W., Day, B.W., Wray, N.R.*, et al.* (2014). NFIB-mediated repression of the epigenetic factor Ezh2 regulates cortical development. J Neurosci *34*, 2921-2930.

Riesenberg, S., Groetchen, A., Siddaway, R., Bald, T., Reinhardt, J., Smorra, D., Kohlmeyer, J., Renn, M., Phung, B., Aymans, P.*, et al.* (2015). MITF and c-Jun antagonism interconnects melanoma dedifferentiation with pro-inflammatory cytokine responsiveness and myeloid cell recruitment. Nat Commun *6*, 8755.

Smith, A.G., Brightwell, G., Smit, S.E., Parsons, P.G., and Sturm, R.A. (1998). Redox regulation of Brn-2/N-Oct-3 POU domain DNA binding activity and proteolytic formation of N-Oct-5 during melanoma cell nuclear extraction. Melanoma Res *8*, 2-10.

Smith, A.G., Lim, W., Pearen, M., Muscat, G.E., and Sturm, R.A. (2011). Regulation of NR4A nuclear receptor expression by oncogenic BRAF in melanoma cells. Pigment Cell Melanoma Res *24*, 551-563.

Thurber, A.E., Douglas, G., Sturm, E.C., Zabierowski, S.E., Smit, D.J., Ramakrishnan, S.N., Hacker, E., Leonard, J.H., Herlyn, M., and Sturm, R.A. (2011). Inverse expression states of the BRN2 and MITF transcription factors in melanoma spheres and tumour xenografts regulate the NOTCH pathway. Oncogene *30*, 3036-3048.

Verfaillie, A., Imrichova, H., Atak, Z.K., Dewaele, M., Rambow, F., Hulselmans, G., Christiaens, V., Svetlichnyy, D., Luciani, F., Van den Mooter, L.*, et al.* (2015). Decoding the regulatory landscape of melanoma reveals TEADS as regulators of the invasive cell state. Nat Commun *6*.
